# Eight port millimeter wave HMSIW MIMO antenna array for V2V communication

**DOI:** 10.1038/s41598-025-24710-4

**Published:** 2025-11-20

**Authors:** Gunjan Srivastava, Amit Kumar, Vimal Kumar, Akhilesh Mohan, Sachin Kumar, Om Prakash Kumar

**Affiliations:** 1 Department of Electronics and Communication Engineering, Graphic Era (Deemed to Be) University, Dehradun, 248002 India; 2 Department of Electronics and Communication Engineering, Indian Institute of Technology Roorkee, Roorkee, 247667 India; 3 Department of Electronics and Communication Engineering, Galgotias College of Engineering and Technology, Greater Noida, 201310 India; 4 Department of Electronics and Communication Engineering, Manipal Institute of Technology, Manipal Academy of Higher Education, Manipal, 576104 India

**Keywords:** Antenna, Eight-port, HMSIW, MIMO, V2V, SDG 9 (Industry, innovation and infrastructure), SDG 11 (Sustainable cities and communities), Optics and photonics, Optical materials and structures

## Abstract

In this paper, an eight-port millimeter-wave multiple-input-multiple-output (MIMO) antenna array operating at 28 GHz for vehicle to vehicle (V2V) communication is designed and developed. It consists of eight 2 × 2 half-mode substrate integrated waveguide (HMSIW) antenna arrays placed along the edges of a regular octagon. The 2 × 2 HMSIW antenna array is fed through a corporate feeding network that comprises of two-stage in-phase T-junction power divider. The designed antenna array has an overall volume of 0.72 ${\lambda }_{0}^{3}$, where *λ*_0_ is the free-space wavelength at operating frequency of 28 GHz. It exhibits excellent peak realized gain (> 12.2 dBi) and high inter-port isolations (> 30 dB) among all the antenna elements without usage of any decoupling network. The MIMO performance of the proposed MIMO antenna array is evaluated in terms of envelope correlation coefficient (ECC) and diversity gain (DG). The performance parameters lie within their acceptable limits. The link budget analysis of the proposed MIMO antenna array is performed with the evaluation of path losses and link margins. To further enhance the gain and radiation characteristics, the 8-port MIMO antenna design, originally based on a 2 × 2 array, is extended to a 4 × 4 array configuration. High gain, high inter-port isolation and excellent radiation characteristics make the designed millimeter-wave MIMO antenna array a potential candidate for V2V communication system.

## Introduction

In recent years, vehicle to everything (V2X) communications have become an indispensable part of intelligent transport system (ITS) and advanced driver assistance systems (ADAS)^1^. It provides the robust communication between the vehicles and nearby entities such as vehicle to vehicle (V2V), vehicle to infrastructure (V2I), vehicle to pedestrian (V2P) and vehicle to network (V2N) on account of its advantages such as improved road safety, reduced traffic congestion and autonomous driving^2^. The 3GPP, in its fifth-generation new radio (5G NR) Release 16, 17 has dedicated the newer standards for V2V based communications^3,4^. As new vehicular applications require the rapid exchange of high-resolution sensor and control data, the existing 5.9 GHz band—allocated from 5.85 to 5.925 GHz under IEEE 802.11p—cannot accommodate the growing demand for additional spectrum. The millimeter-wave frequency bands are being explored in the recent years to overcome the growing requirement of high data rates for V2V communication. The channel propagation models in different scenarios such as urban, semi-urban, and highway conditions are explored for millimeter-wave bands and 28, 38, 60, 76 GHz are found to be potential frequencies for V2V communications^5^. The millimeter-wave bands offer high data rate, lower latency and highly reliable wireless communication channel. Compared to other millimeter-wave bands, the 28 GHz is less prone to atmospheric attenuations^6,7^. This frequency band, which is commercially used for 5G cellular communications and falls within the millimeter-wave spectrum, is being increasingly explored for future V2V applications due to its abundant and underutilized bandwidth^8–10^. Moreover, high throughput for V2V communications can be attained with the deployment of multiple-input-multiple- output (MIMO) antennas without any increase in power level^11,12^.

Several two-element^13–17^, four-element^18–21^, and higher order (n ≥ 6)^22–25^, MIMO antenna systems for millimeter-wave bands are reported in the literature. In^13^, two-element MIMO antenna radiating at 29 GHz having the slits in the radiators with a gain of 6 dBi is presented. The slits enable a good isolation between the radiating elements. A high gain two-element metamaterial-based MIMO antenna at 28 GHz is designed and developed in^14^. Another electromagnetic bandgap (EBG) structure based two-element MIMO antenna at 28 GHz is reported^15^. In^16^, two-element 3D printed dielectric lens integrated MIMO antenna with improved gain for millimeter-wave applications is presented. A substrate-integrated waveguide (SIW) cavity backed millimeter-wave filtering patch antenna is presented in^17^. In^18^, a four-element ring-shaped MIMO antenna at 28 GHz is reported that utilizes ground plane decoupling structure for isolation improvement. The defected ground structures are deployed for isolation enhancement in^19^. In^20^, a four-element circular patch with a rectangular slot etched MIMO antenna for Ka-band is designed and developed. In^21^, a four-element MIMO antenna with split ring resonator (SRR) and end-fire characteristics is presented. Higher order (n ≥ 6) MIMO antennas with limited gains are designed and developed for higher data rate requirements at lower millimeter-wave frequencies^22–25^. In^25^, a sub-6 GHz and millimeter-wave 5G V2X MIMO antenna array is designed and developed.

In a nutshell, the aforementioned MIMO antennas deploy decoupling network/structures, such as metamaterials, EBG, defected ground structure (DGS), parasitic elements, for isolation improvement and most of them have limited gains. To overcome the gain limitations, MIMO antenna arrays are one of the promising solutions. In recent years, two- and four-element MIMO antenna arrays are reported in the literature^26–30^. In^26^, a two-port MIMO antenna array for 38 GHz with good inter-port isolation is designed, where each port comprises of a four-element corporate fed antenna array. In^27^, a two-element 2 × 1 diamond-shaped MIMO antenna array for 5G millimeter-wave system is designed. High isolation between the array elements is observed due to their orthogonal placement. A four-port millimeter-wave MIMO antenna array at 28 GHz with a gain 7.9 dBi is reported in^28^. In^29^, a four-port MIMO antenna array for 28 GHz with good inter-port isolations and high gain is presented. Each port of these structures comprises of four radiating elements, which is fed through two T-junction power dividers. A four-element dielectric resonator MIMO antenna array with inter-port isolations of 15 dB at 28 GHz is reported^30^. However, the higher data rates are essential for V2V millimeter-wave communications such as autonomous driving and real-time traffic management to ensure reliable and robust communication.

In this paper, an eight-port MIMO antenna array for 28 GHz V2V millimeter-wave communication is presented. The unit element array of the designed antenna system consists of four half-mode substrate integrated waveguide (HMSIW) cavity resonators that are arranged in 2 × 2 array pattern, which are fed through a two-stage in-phase T-junction power divider. The unit element arrays are placed across the edges of the regular octagon. The designed MIMO antenna array possesses high gain and high inter-port isolations at the operating frequency of 28 GHz. The designed MIMO antenna array exhibits excellent MIMO performance. Building upon the 2 × 2 array design, the eight-port MIMO antenna array is expanded into a 4 × 4 configuration to achieve improved gain and radiation properties.

## Antenna configuration

**Fig. 1:**
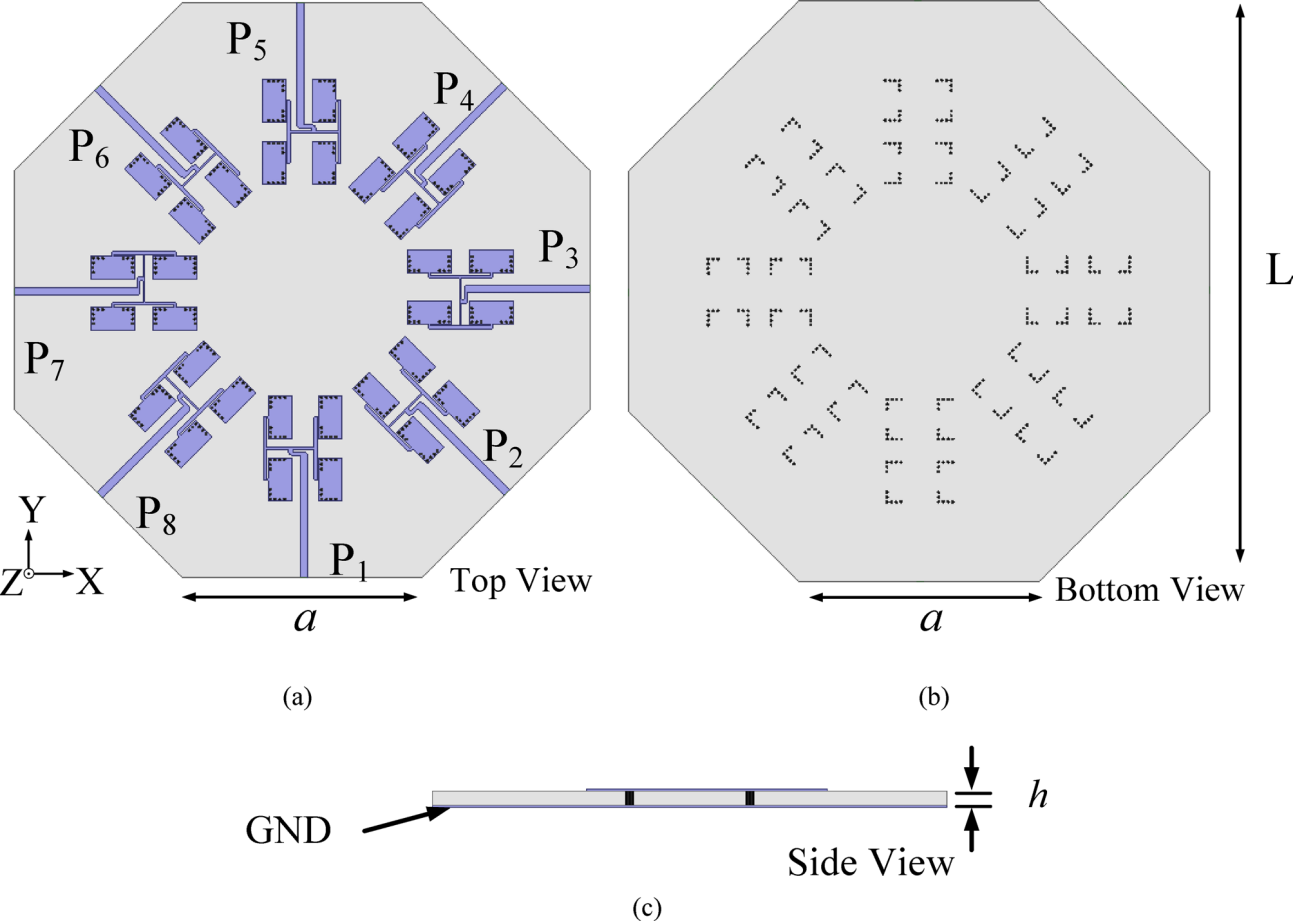
Proposed eight-port millimeter-wave MIMO antenna array: (**a**) Top view, (**b**) Bottom view, (**c**) Side view [*L* = 61 mm, *a* = 25.25 mm, *h* = 0.254 mm].

Figure 1 shows the proposed millimeter-wave MIMO antenna array for V2V communication systems. It operates in n257 band of 5G spectrum. It is designed on 0.254 mm thick low loss high frequency RT/duroid 5880 substrate with dielectric constant of 2.2 and loss tangent of 0.0008. It comprises of eight HMSIW antenna arrays, which are arranged across the edges of a regular octagon of dimension *a*. Each corporate-fed HMSIW antenna array with four radiating elements is arranged in 2 × 2 array pattern. The corporate feeding network comprises of two-stage T-junction power divider with in-phase characteristics. The two-stage T-junction power divider is designed to divide the input power equally among all four radiating HMSIW elements across each antenna array. Quarter-wave (*λ*/4) transformers are deployed at each T-junction to obtain good impedance match.

## Design methodology

In this section, the design methodology of the proposed millimeter-wave MIMO antenna array for V2V communication system is discussed in detail along with its working principle.

### HMSIW cavity resonator

SIW cavity resonators are popularly used nowadays for the design of high-performance microwave and millimeter-wave components due to its inherent properties such as low loss, low cost, good efficiency, low mutual coupling and its ease of integration with other planar components^31^. A full-mode SIW (FMSIW) cavity can be bifurcated into two HMSIW cavity by bisecting it along the fictitious symmetrical magnetic wall. Due to this bifurcation, HMSIW cavity not only possess the original field distribution but also results in size miniaturization^32^, which is used for design of several different types of miniaturized microwave MIMO antennas^33–36^.

**Fig. 2:**
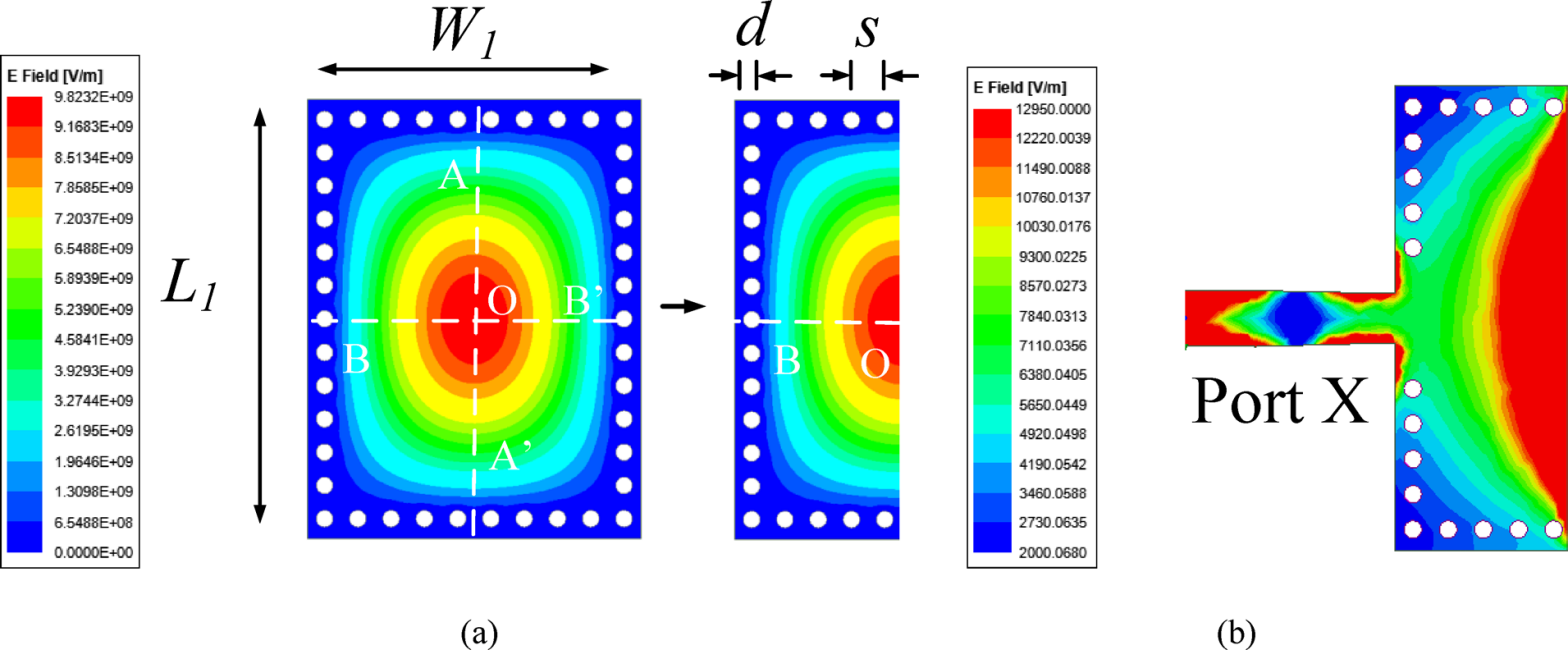
Evolution of HMSIW antenna along with its electric field distributions in the dominant TE_110_ mode: (**a**) For FMSIW and HMSIW cavity resonator, (**b**) HMSIW antenna with microstrip feed (*L*_1_ = 6.1 mm, *W*_1_ = 4.5 mm, *d* = 0.25 mm, *s* = 0.50 mm).

The evolution of microstrip-fed HMSIW cavity resonator is shown in Fig. 2. At first, a FMSIW cavity resonator of dimensions *L*_1_ × *W*_1_ that supports dominant TE_110_ mode at 28 GHz is designed. The resonant frequency of FMSIW cavity in TE_110_ mode is determined through the following expression^37^:

1 $${f}_{{TE}_{110}}=\frac{c }{2\sqrt{{\varepsilon }_{reff}}}\sqrt{{\left(\frac{1}{{L}_{eff}}\right)}^{2}+{\left(\frac{1}{{W}_{eff}}\right)}^{2}}$$

where *c* is the speed of the light in free-space, ${\varepsilon }_{reff}$ is the effective dielectric constant, ${L}_{eff}$ and ${W}_{eff}$ are the effective dimensions of the equivalent resonant cavity and can be evaluated using equations provided in^38^.

2 $${L}_{eff}=L-1.08\frac{{d}^{2}}{s}+0.1\frac{{d}^{2}}{W}$$

3 $${W}_{eff}=W-1.08\frac{{d}^{2}}{s}+0.1\frac{{d}^{2}}{W}$$

Here, diameter *d* and via spacing *s* are chosen such that there is confinement of fields inside FMSIW cavity resonator and they must satisfy the following conditions:

4 $$d< \frac{{\lambda }_{g}}{5},s\le 2d$$

where ${\lambda }_{g}$ is the guided wavelength of the propagating wave in SIW structure^32^.

5 $${\lambda }_{g}=\frac{{\lambda }_{0}}{\sqrt{{\varepsilon }_{r}-{\left(\frac{{\lambda }_{0}}{{\lambda }_{c}}\right)}^{2}}}$$

where ${\lambda }_{0}$ is the operating wavelength and ${\lambda }_{c}$ is the cut-off wavelength of the wave.

**Fig. 3:**
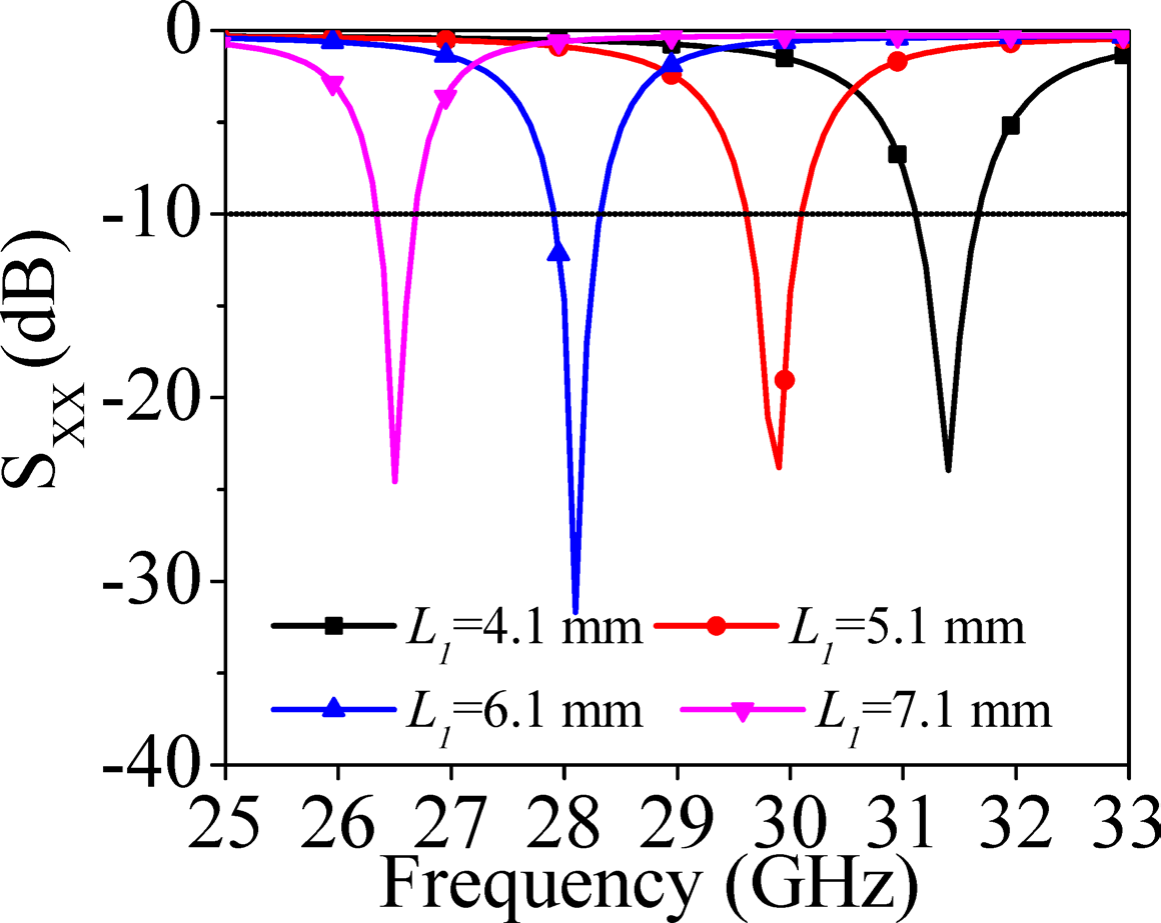
Simulated S-parameters of microstrip fed HMSIW antenna shown in Fig. 2b for different values of *L*_1_.

Figure 2a shows E-field distribution for TE_110_ mode at 28 GHz. As E-fields are symmetrical along the principal plane AA’, they can be bifurcated into two HMSIW cavity resonators by applying magnetic wall concept. The HMSIW cavity resonator possesses the similar modal characteristics as that FMSIW cavity resonator at 28 GHz. Further, the HMSIW cavity resonator is fed through a microstrip feed line as shown in Fig. 2b. Figure 3 demonstrates the simulated S-parameters of HMSIW antenna [Fig. 2b] for different values of *L*_1_. As the value of *L*_1_ increases, the resonant frequency shifts towards the lower value. This variation of *L*_1_ facilitates to design the antenna systems for different frequencies of lower millimeter-wave spectrum.

### Feed network

**Fig. 4:**
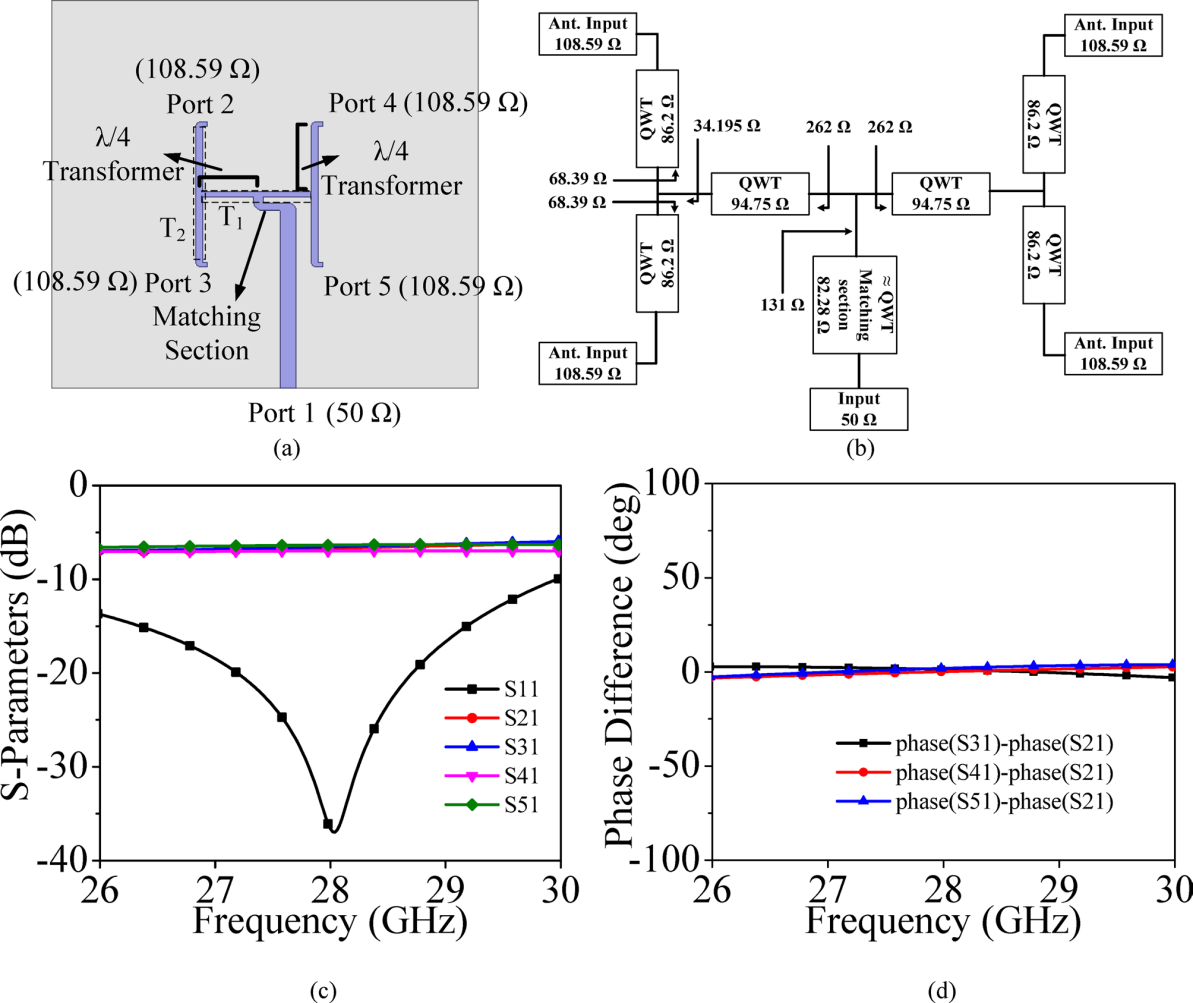
(**a**) Configuration of the feed network, (**b**) Impedance transformation block diagram, (**c**) Its simulated S-parameters, (**d**) Phase difference.

To design the proposed high gain MIMO antenna array, a corporate feed network is designed. Figure 4a and b show the configuration and impedance transformation block diagram at each stage of the two-stage T-junction power divider based corporate feed network, respectively. It consists of three stages: matching section and two-stages of *λ*/4 transmission line-based T-junction power divider. Here, Port 1 is the input port, while Port 2, Port 3, Port 4 and Port 5 are the output ports. The characteristics impedance of the input port (Port 1) is 50 Ω, and the impedances of the other remaining ports are 108.59 Ω, which are the input impedances of the HMSIW cavities. The feed network was accordingly designed using a cascade of quarter-wave transformers (QWTs) to provide an efficient and broadband impedance transformation from 50 Ω to 108.59 Ω. The specific QWT impedance values (86.2 Ω, 94.73 Ω, and 82.28 Ω) are chosen using the standard transformation relation, *Z*_*QWT*_ = $\sqrt{{Z}_{1}{Z}_{2}}$, ensuring smooth impedance tapering and minimal reflection. The matching section is used to match the 50 Ω of the input port to 131 Ω input impedance of a T-junction *T*_1_. The T-junction *T*_1_ power divider divides the power into two equal halves using two quarter-wave transformers. The output power from the T-junction *T*_1_ is further divided into two equal halves using another T-junction *T*_2_, which also utilizes the quarter wave transformation. For clarity, the complete impedance transformation path—including all matching stages and transformer values is illustrated in the impedance block diagram [Fig. 4b]. The S-parameters for the two-stage T-junction power divider are plotted in Fig. 4c. The values of S_21_, S_31_, S_41_ and S_51_ are − 6.70 dB, − 6.54 dB, − 6.98 dB and − 6.34 dB, respectively, demonstrates nearly equal power divisions at the output ports. The reflection coefficient at Port 1 is better than 36 dB. Figure 4d shows the phase differences between the different ports of the two-stage T-junction power divider and the phase differences are nearly same at the operating frequency of 28 GHz.

### Unit element array

**Fig. 5:**
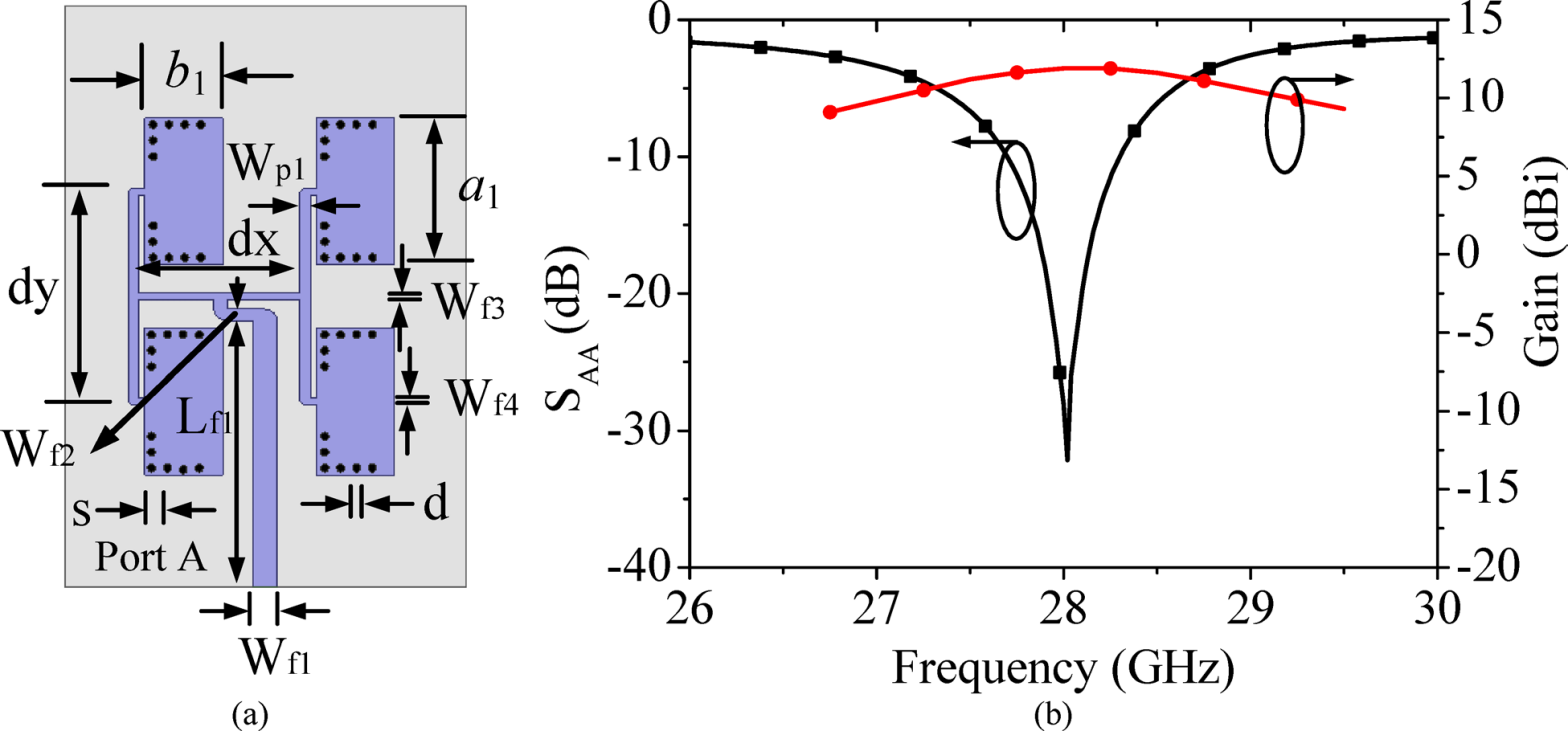
(**a**) Unit element array along with its, (**b**) Simulated S_AA_-parameters and gain [*a*_1_ = 5.598 mm, *b*_1_ = 2.45 mm, *L*_*f*1_ = 8.344 mm, *W*_*f*1_ = 0.775 mm, *W*_*p*1_ = 0.327 mm, *dx* = 5.1 mm, *dy* = 6.41 mm, *s* = 0.25 mm, *d* = 0.50 mm].

**Fig. 6:**
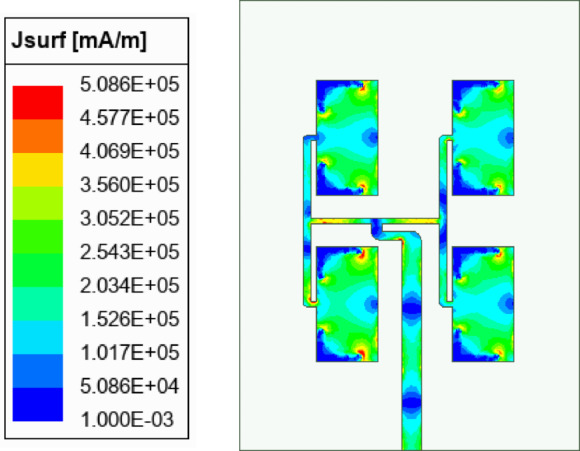
Surface current distribution of the UE array at 28 GHz.

To develop the proposed high gain MIMO antenna array, a unit element antenna (UEA) array at 28 GHz is designed using the two stage T-junction power dividers as designed in the above sub-section. The configuration and dimensions of UE array is shown in Fig. 5. Four HMSIW cavity resonators of dimensions *a*_1_ × *b*_1_ are placed in 2 × 4 array pattern. The HMSIW cavity resonators are excited through a corporate feed network. With the usage of two equal split T-junction power dividers, the input power is equally in-phase divided among all the four HMSIW cavity resonators. The *λ*/4 impedance transformers are deployed at each T-junction for good impedance matching at each port of the corporate feed network. The separation between the HMSIW resonators across *x*- and *y*-axes are chosen as *dx* and *dy*, respectively. The larger values of *dx* and *dy* will result in side-lobes while the smaller values will result in difficulties in the arrangement of the feed network. As the trade-off between these two, the values of *dx* and *dy* are optimally chosen as 5.1 mm and 6.41 mm, respectively, which is approximately 0.476*λ*_0_ and 0.58*λ*_0_ at the operating frequency of 28 GHz. Figure 5b shows the simulated S_AA_-parameters and peak realized gain of the unit element array as depicted in Fig. 5a. It radiates at 28 GHz with a high peak realized gain of 12.2 dBi. The designed UE array will be used to design the proposed eight-port millimeter-wave MIMO antenna array. The surface current distribution at the operating frequency of 28 GHz is plotted in Fig. 6. It can be observed from the figure that the nearly equal amount of surface currents is present at each of the HMSIW antenna element, which demonstrates equal power distribution at its operating frequency.

### Proposed eight-port millimeter-wave MIMO antenna array

**Fig. 7:**
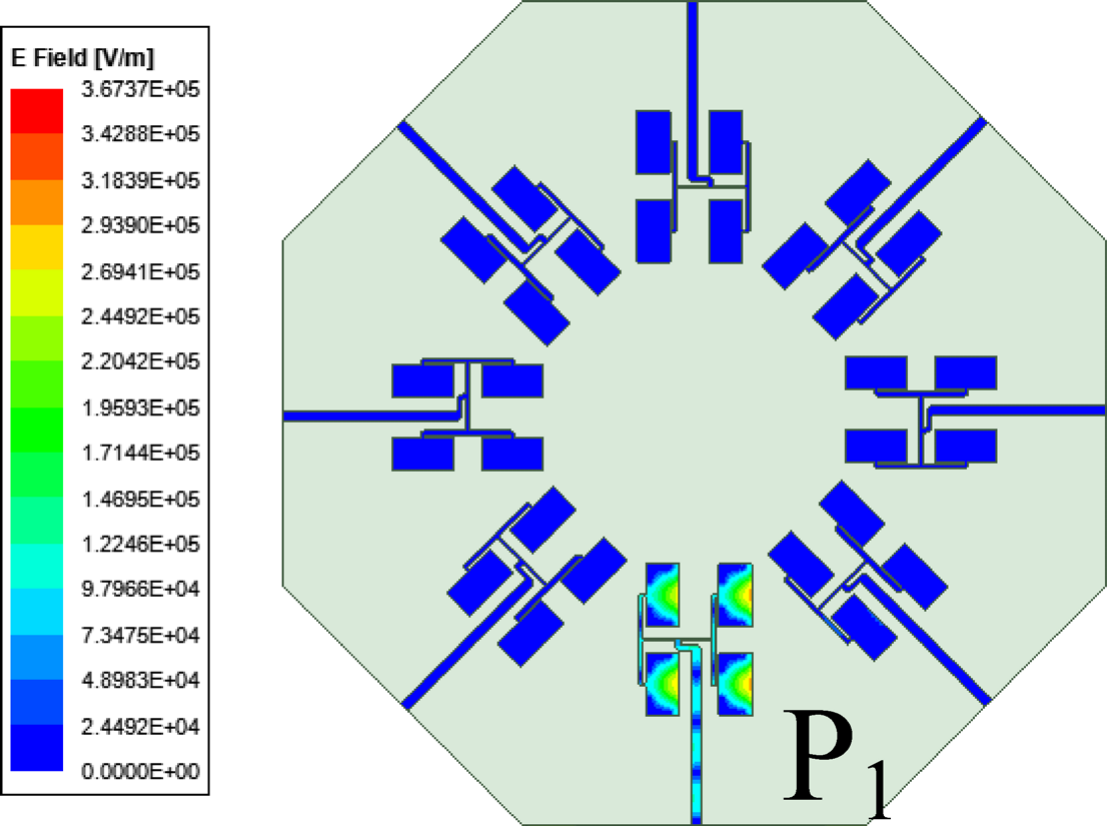
Electric field distribution under Port P_1_ excitation at 28 GHz.

To design the proposed eight-port millimeter-wave MIMO antenna array, eight-unit element arrays designed in above sub-section are placed across the edges of a regular octagon of dimension *a* as shown in Fig. 1. The minimum separation between the unit element array is 1.33 mm, which is approximately 0.12*λ*_0_, where *λ*_0_ is the free space wavelength at 28 GHz. The design parameters of the proposed MIMO antenna array are presented in the captions of Fig. 1. The center-to-center spacing between a 2 × 2 subarray and its adjacent 2 × 2 subarray is approximately 12.8 mm, which is 1.19*λ*_0_, where *λ*_0_ is the free-space wavelength at the operating frequency of 28 GHz. The minimum spacing between the elements of a 2 × 2 subarray and the elements of its adjacent 2 × 2 subarray is approximately 1.32 mm, which 0.12*λ*_0_. This minimum spacing not only facilitates sufficient space for the connectors but also ensures the undistorted radiation patterns of the individual 2 × 2 subarrays. High inter-port isolations in the proposed work can be attributed towards the strategic placement of unit element arrays across the edges of the regular octagon. Figure 7 demonstrates the electric field distribution for Port P_1_ excitation along with its scale. It is observed from the figure that if one of the ports (say P_1_) is excited, the unit elements of array radiate through TE_110_ mode of HMSIW cavity resonator at 28 GHz. Moreover, there is negligible fields are present at the other ports (P_2_, P_3_, …P_8_). They are mainly concentrated in the unit element array that results in high inter-port isolation.

### Design steps and flowchart

**Fig. 8:**
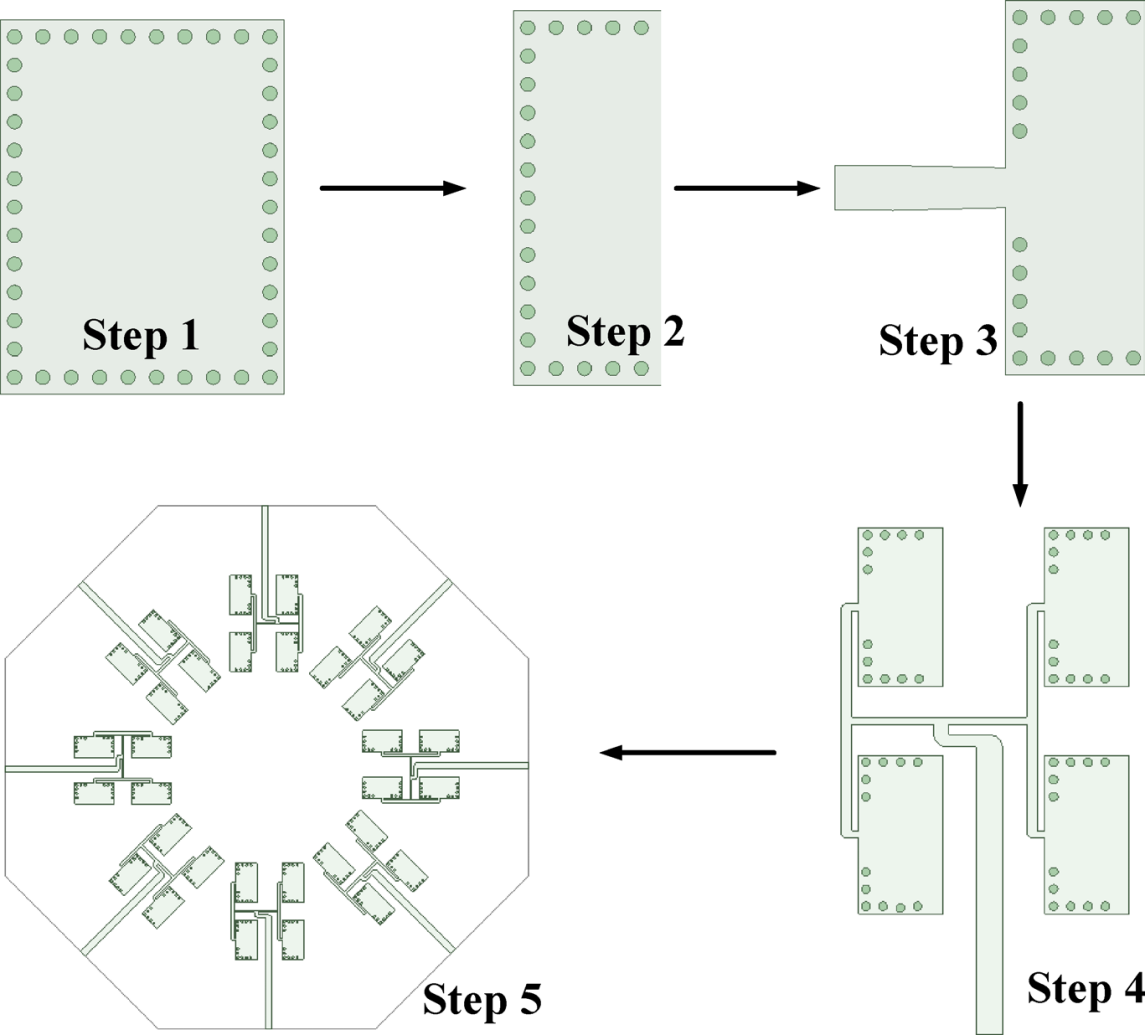
Step-by-step development stages of the proposed MIMO antenna array.

**Fig. 9:**
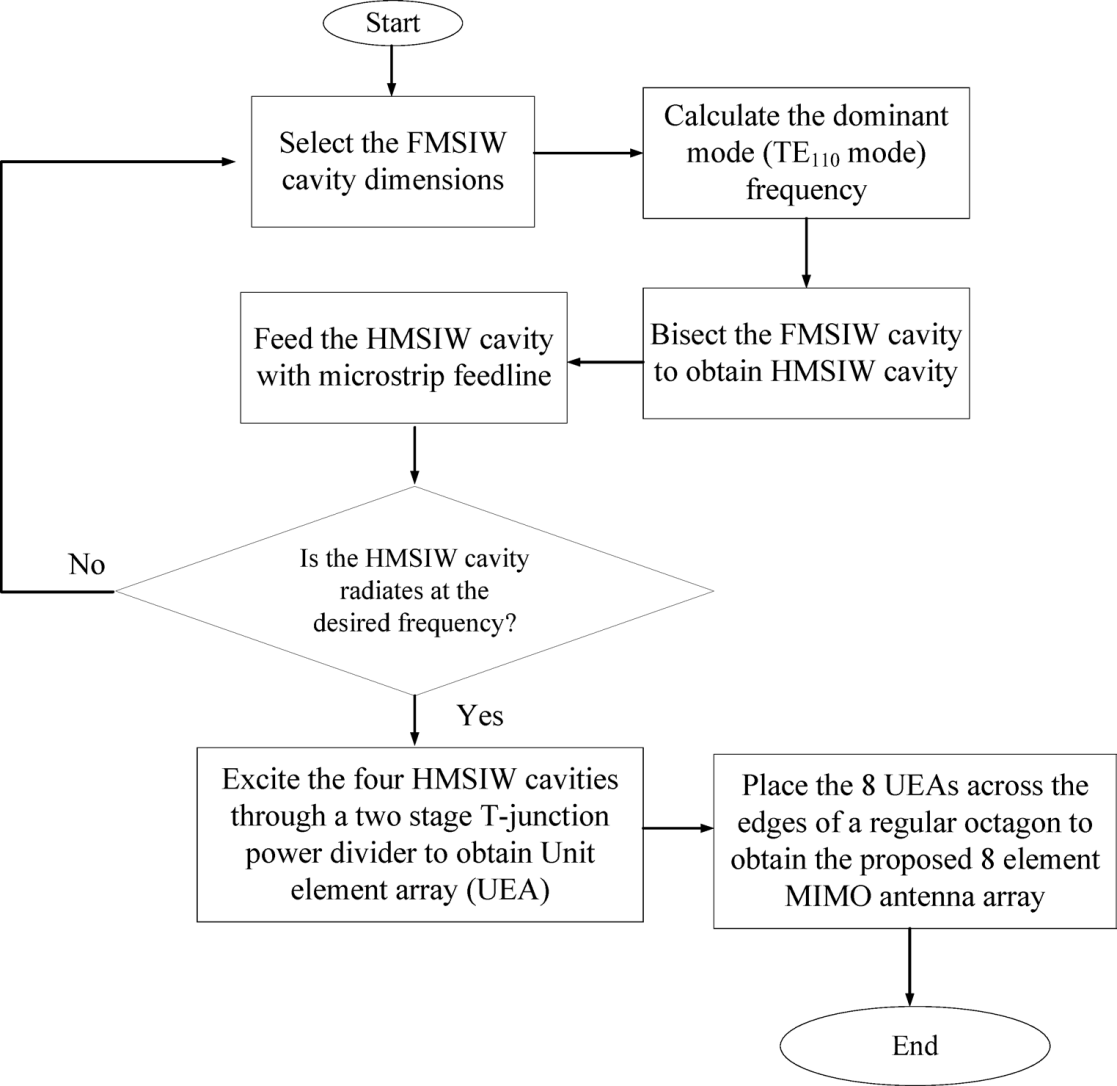
Flowchart of the proposed millimeter-wave MIMO antenna array.

The step-by-step design process of the proposed millimeter-wave MIMO antenna array along with its schematic flowchart are shown in Figs. 8 and 9. Initially, in Step-1, a FMSIW cavity with TE_110_ mode as the dominant mode, operating at frequency *f*_0_, is selected. Subsequently, the FMSIW cavity is bisected using the magnetic wall concept to obtain the HMSIW in Step 2. In Step 3, the dominant TE_110_ mode of HMSIW cavity is excited through microstrip feed lines. There is a slight shift in operating frequency is observed, which can be fine-tuned by varying the dimensions of the HMSIW cavity. Further, to enhance the gain and radiation characteristics of the HMSIW antenna, a 2 × 2 UEA array is designed by feeding the four HMSIW cavity resonators through a two stage T-junction power divider in Step 4. Finally, eight-UEA array, designed in Step-4, are placed across the edges of a regular octagon to obtain the proposed eight-port millimeter-wave MIMO antenna for V2V communications. This step-by-step design process incorporates the development stages of the proposed MIMO antenna array system.

A comprehensive and step-wise explanation of MIMO system simulations is presented in this section. Initially, the full-wave electromagnetic simulation for the proposed eight-port millimeter-wave HMSIW antenna array is performed using 3D EM solver Ansys HFSS to extract its S-parameters [S_ii_, (*i* = 1 to 8) and S_ij_ (*i* ≠ *j*, *i*, *j* = 1 to 8)] and far-field radiation patterns of each port at the desired operating frequency of 28 GHz. The simulated results are then imported into MATLAB for further post-processing. The envelope correlation coefficient (ECC) is calculated, either using the S-parameters or, more accurately, from the far-field patterns, to assess the degree of independence between antenna elements. From the ECC, the diversity gain (DG) is derived, indicating the robustness of the system in multipath environments. Furthermore, to examine the communication capacity of the proposed V2V millimeter-wave MIMO antenna array, the link budget analysis is performed for two different scenarios, line of sight (LOS) and non-LOS (NLOS), at its operating frequency. The studies for path losses and link margins of the proposed antenna system are performed using channel model provided in the open literature. For brevity, the communication between the antenna systems installed over the roof-top of the vehicles can be considered. The MIMO system simulations and examination of communication capacity provide a comprehensive insight into the MIMO system’s performance, validating its suitability for high data rate, low-correlation, and high reliability applications in next-generation vehicular communication systems.

## Results and discussion

**Fig. 10:**
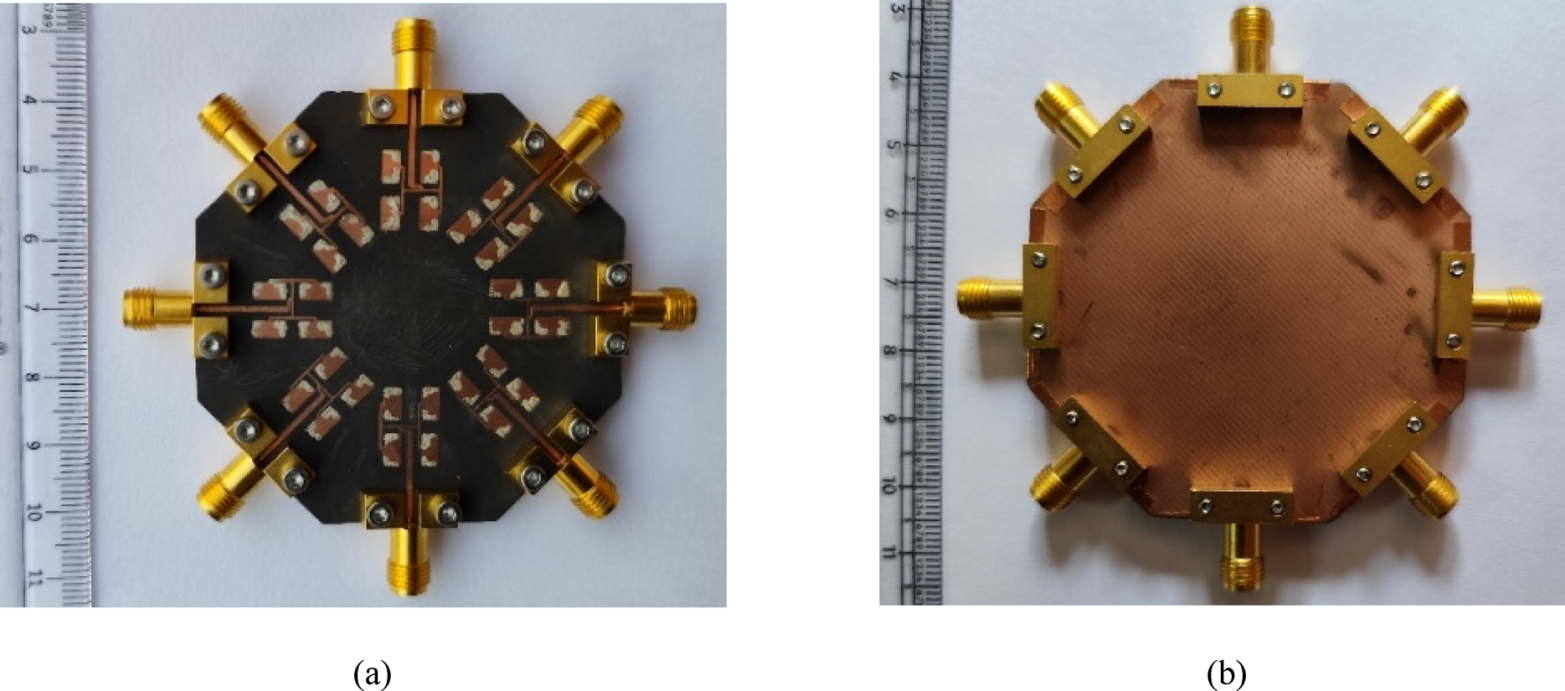
Fabricated prototype of the proposed eight-port millimeter-wave MIMO antenna array: (**a**) Top view, (**b**) Bottom view.

**Fig. 11:**
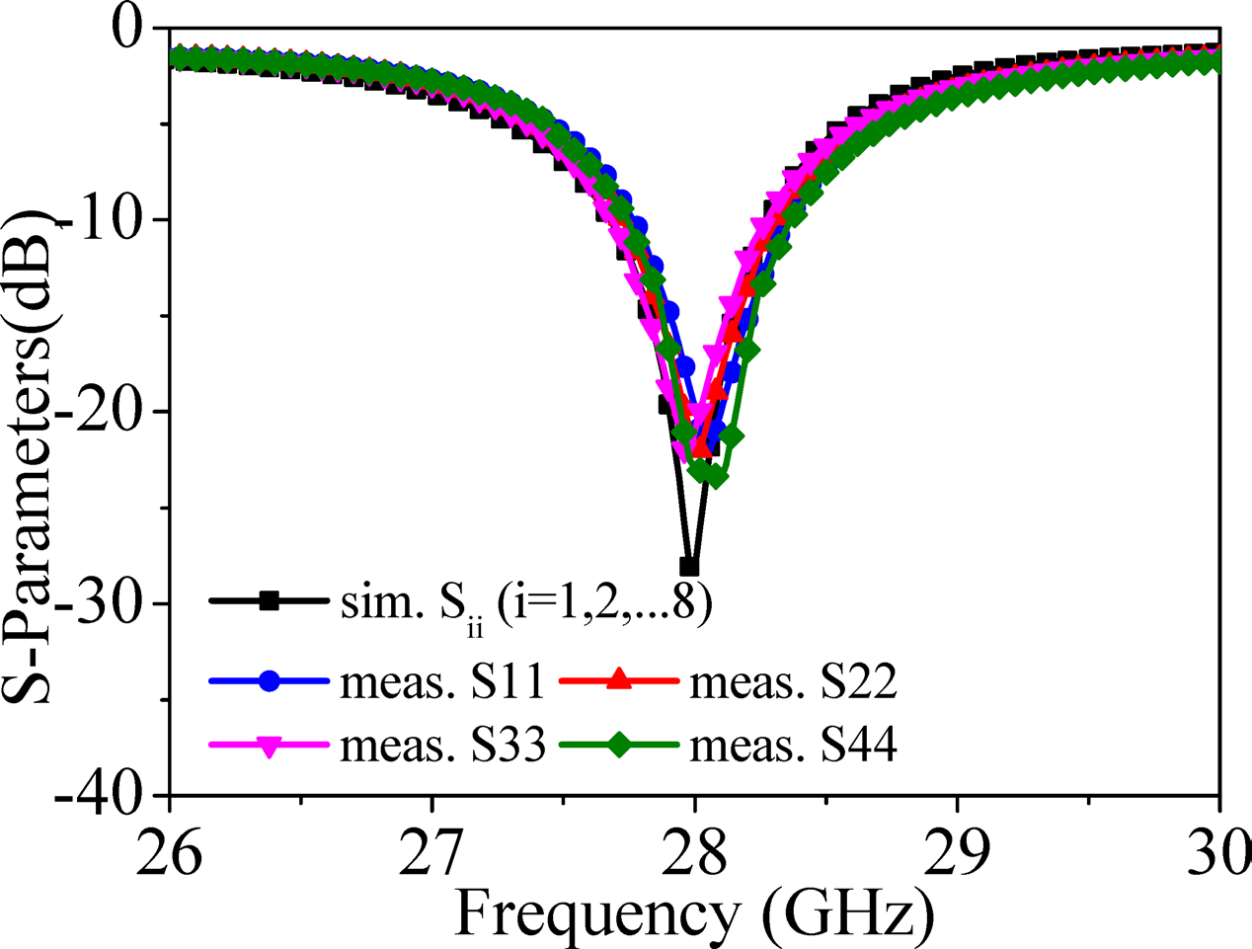
Simulated and measured S_ii_-parameters of the proposed millimeter-wave MIMO antenna array.

**Fig. 12:**
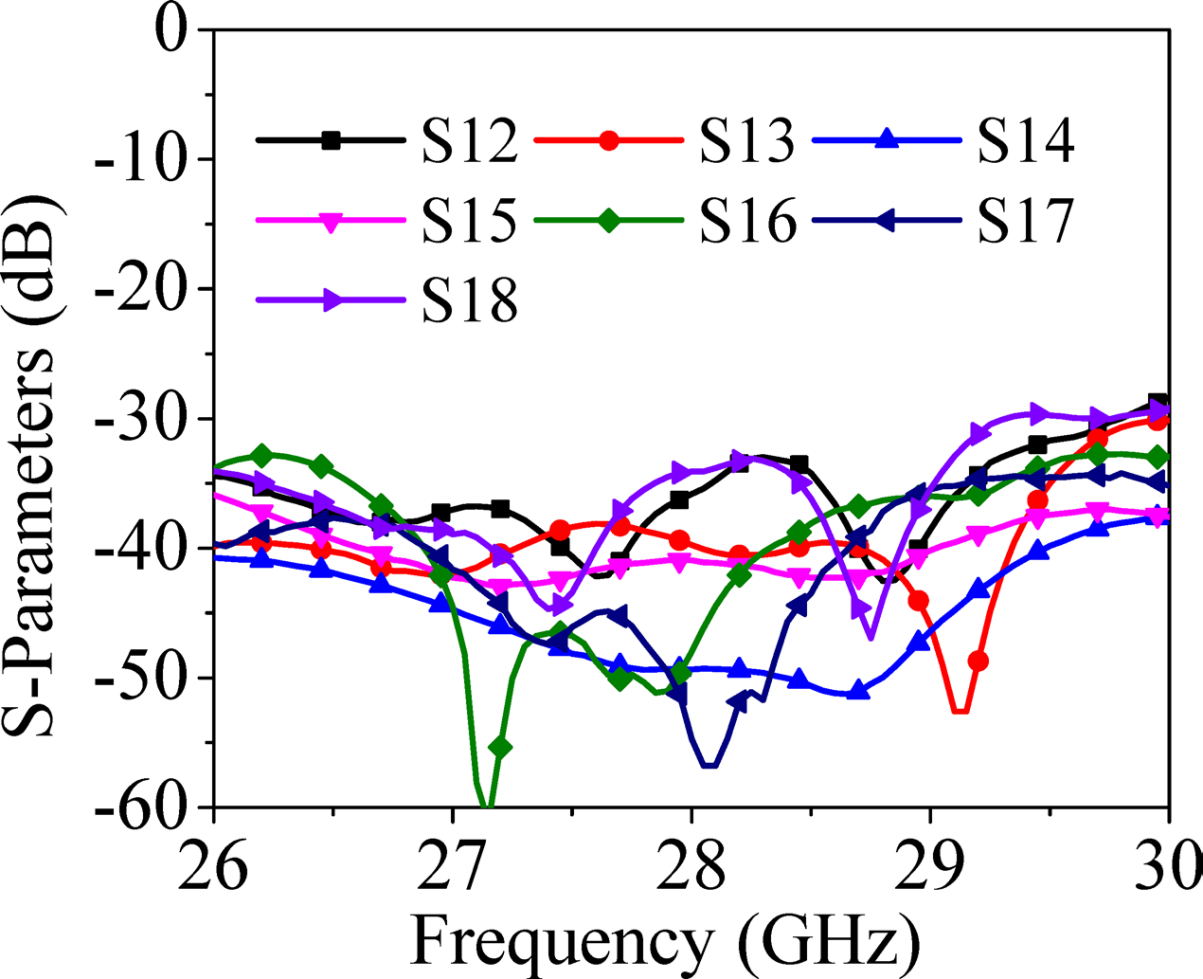
Measured S_ij_-parameters (*i* ≠ *j*) of the proposed millimeter-wave MIMO antenna array.

A prototype of the designed MIMO antenna array is fabricated on 0.254 mm thick RT/duriod 5880 using standard PCB fabrication techniques. The vias are drilled using LPKF Protomat machine and are filled with copper wires of diameter 0.25 mm and silver epoxy paste are applied at the top and bottom sides of the structures for connectivity. As the substrate is very thin (thickness = 0.254 mm), an octagonal-shaped (same as that of the substrate) copper plate is connected at the backside of the antenna system to provide the strength to the structure. Figure 10 shows the photograph of the top and bottom views of the fabricated prototype. Eight 02K243-40ME3 Rosenberger RF connectors are connected at the ports for the measurements. S-parameters of the prototype are measured with the help of Agilent 5247A vector network analyzer (VNA). Figure 11 shows the simulated and measured S_ii_-parameters of the proposed MIMO antenna array. The simulated operating frequency is 28 GHz while there is a slight shift in the measured operating frequencies. The measured inter-port isolations are plotted in Fig. 12 and are better than 30 dB among all the ports. There are some discrepancies between the simulated and measurement results, which can be attributed to several factors, such as connector losses, measuring environmental setup and fabrication tolerances, where even slight dimensional inaccuracies can lead to performance shifts especially at millimeter-wave frequencies. Despite these discrepancies, the measured results follow the simulated ones, confirming the validity of the proposed design.

**Fig. 13:**
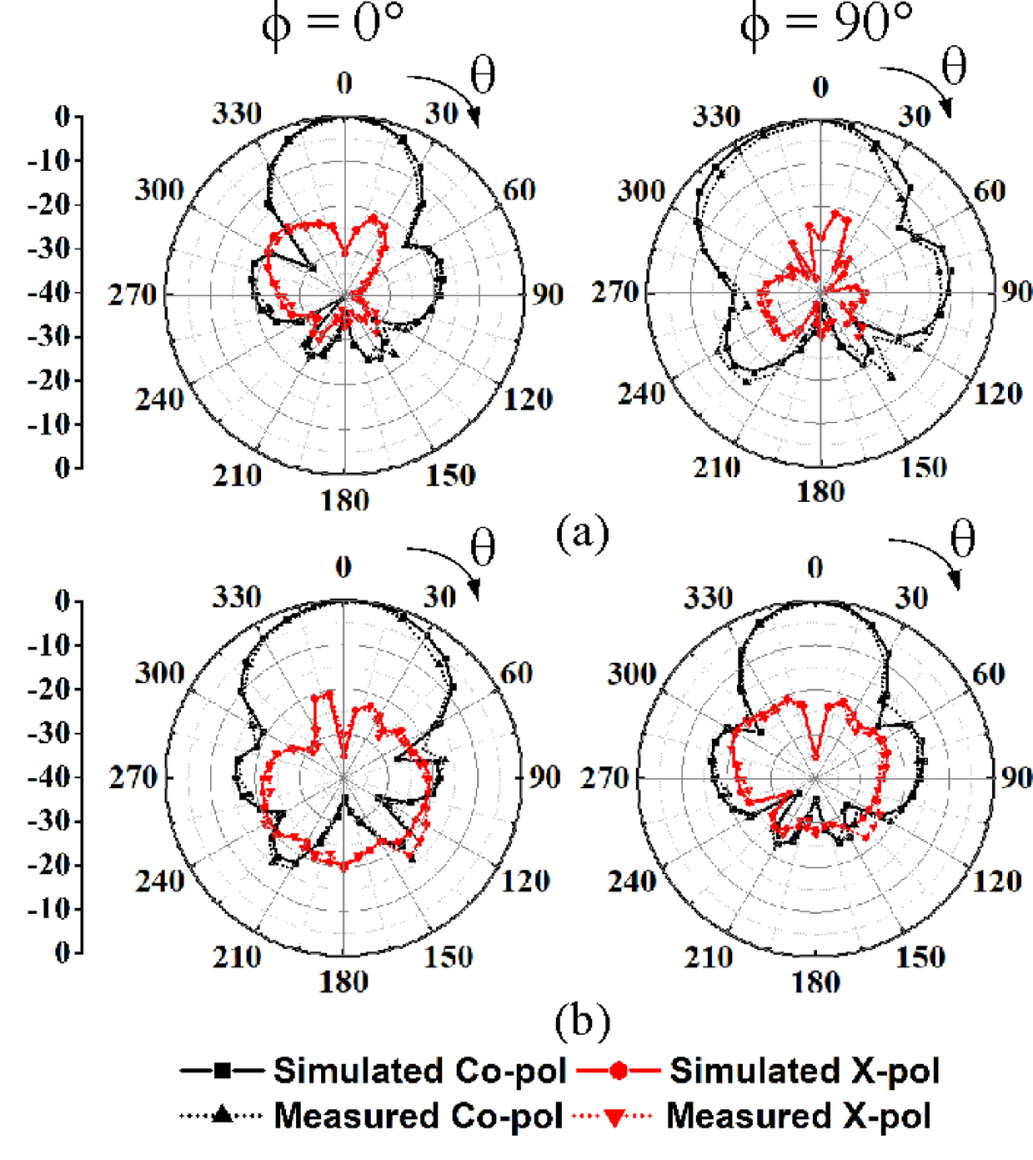
Simulated and measured far-field radiation patterns: (**a**) Port P_1_ excitation in *ϕ* = 0° and *ϕ* = 90° planes, (**b**) Port P_3_ excitation in *ϕ* = 0° and *ϕ* = 90° planes.

**Fig. 14:**
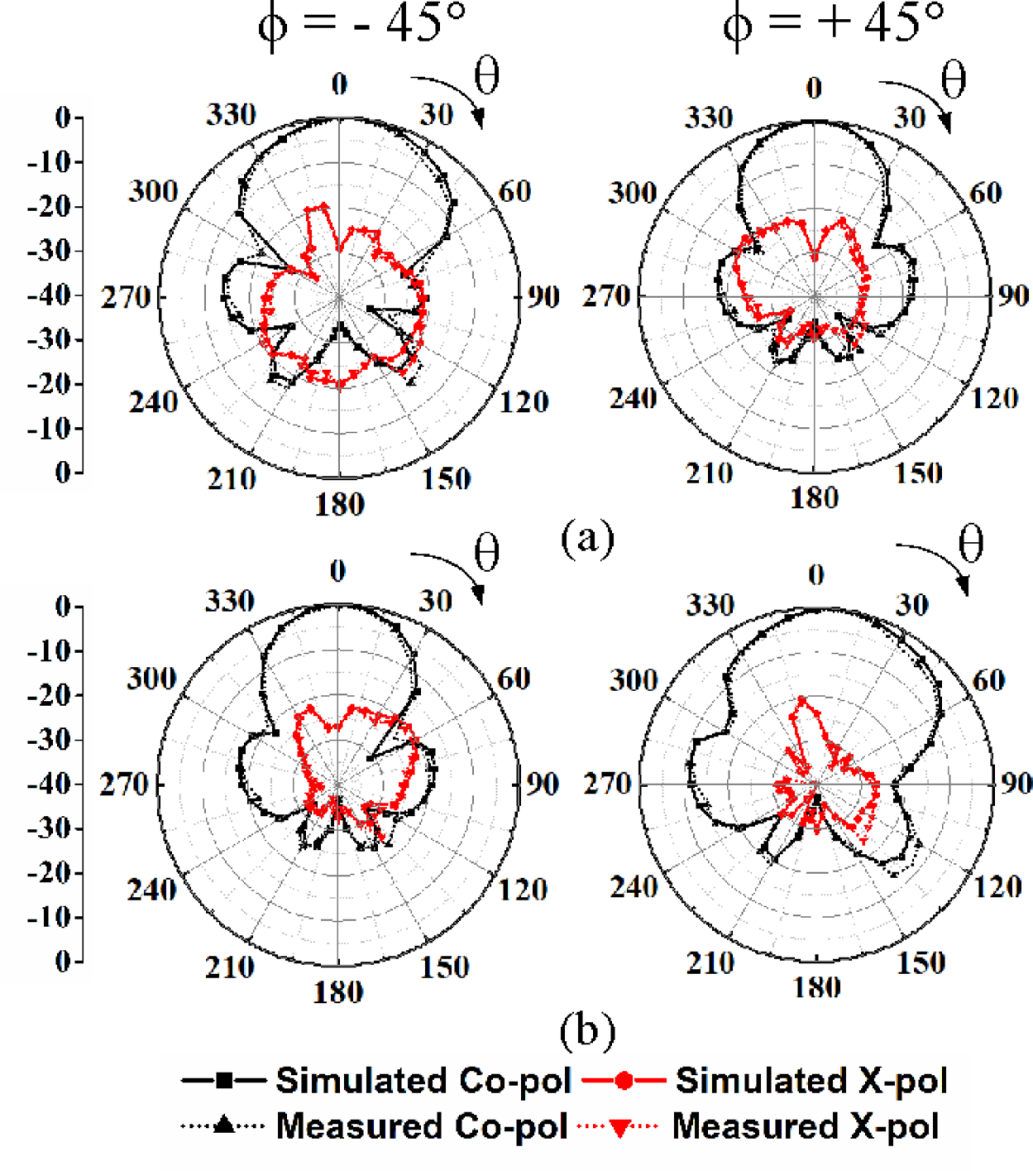
Simulated and measured far-field radiation patterns: (**a**) Port P_2_ excitation in *ϕ* =  −45° and *ϕ* =  +45° planes, (**b**) Port P_4_ excitation in *ϕ* =  −45° and *ϕ* =  +45° planes.

The far-field radiation pattern of the proposed MIMO antenna array is measured in an anechoic chamber. For brevity, the radiation patterns under Port P_1_-P_4_ excitations are provided. Figure 13 depicts the simulated and measured radiation patterns at the operating frequency of 28 GHz for Port P1 and P3 excitations at *ϕ* = 0° and *ϕ* = 90° planes. Whereas, Fig. 14 shows the simulated and measured radiation patterns for Port P2 and P4 excitations at *ϕ* =  −45° and *ϕ* =  +45°, respectively. In these cases, the cross-polarization levels are approximately 25 dB lower than their co-polarization counterparts, with high front-to-back ratio (FTBR) of 30 dB along the broadside direction. The distinct beam tilting is observed in the radiation patterns of some ports in the proposed eight-port HMSIW MIMO antenna array, which results from a combination of structural and electromagnetic factors. The inherent asymmetry of the HMSIW configuration leads to unbalanced field distributions, which naturally steer the radiation away from the boresight. Additionally, the octagonal geometry and close proximity of elements introduce mutual coupling and edge effects that disturb the current distribution, further contributing to the tilt. Moreover, these factors contribute to beam tilting, which can be advantageously utilized for enhancing spatial and polarization diversity in MIMO-based vehicular communication systems.

**Fig. 15:**
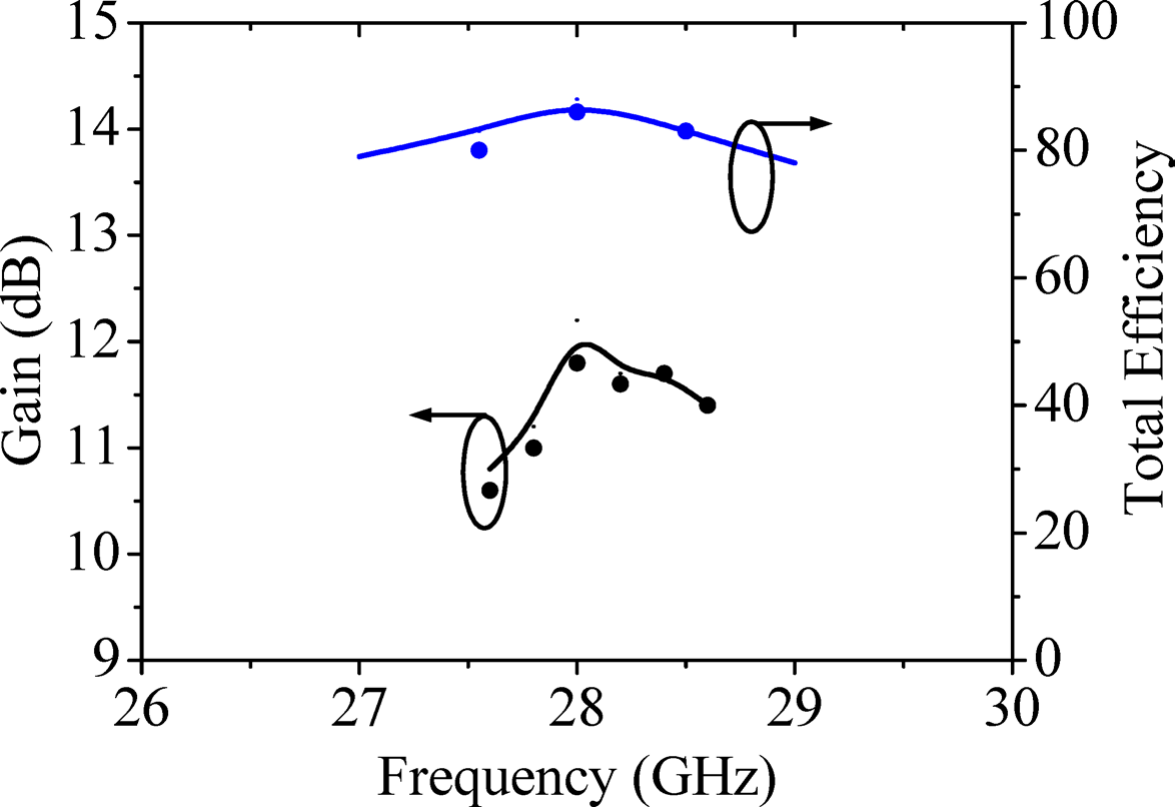
Simulated (solid line) and measured (dotted line) peak realized gain and total efficiency of the proposed millimeter-wave MIMO antenna array.

Figure 15 shows the simulated and measured peak realized gains of the proposed millimeter-wave MIMO antenna array. The simulated peak realized gain is approximately 12.2 dBi, while its measured value is 12 dBi at the operating frequency of 28 GHz. Figure 15 also depicts the total efficiency of the proposed MIMO antenna array and it is better than 80% across the entire operating band. In the present work, the polarization of the HMSIW antenna element is linear due to the excitation of TE_110_ mode. The TE_110_ mode directs the electric field across the open-ended edge of the HMSIW cavity resonator that results in linearly polarized radiations. The HMSIW antenna element is further utilized to design the UEA array of the proposed MIMO antenna array. Since each HMSIW antenna element exhibits linear polarization, when these elements are arranged in a 2 × 2 array pattern to form a UEA array, the overall UEA polarization remains linear, which is aligned along the E-field direction of the individual elements.

## MIMO performance and vehicular integration

### MIMO performance

**Fig. 16:**
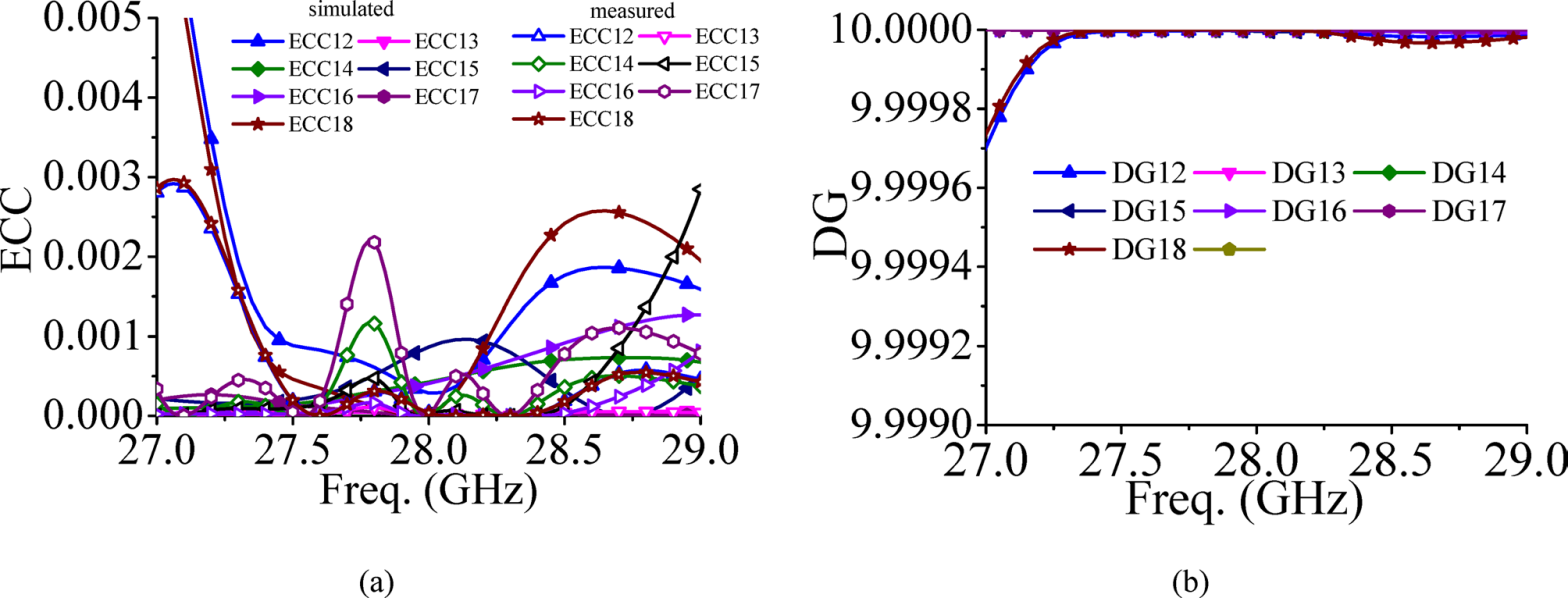
(**a**) Simulated and measured diversity parameters: (**a**) ECC, (**b**) DG.

The MIMO characteristics of the designed antenna system is evaluated in terms of ECC and diversity gain (DG). The simulated ECCs are evaluated from the far field components^39^, whereas measured ECC results are evaluated from the measured S-parameters. Figure 16 shows the ECC and DG values of the proposed millimeter-wave MIMO antenna array. The ECCs are well below their acceptable limits of 0.5, while DGs are nearly 10, which guarantees the excellent MIMO characteristics^40^.

### Vehicular Integration

**Fig. 17:**
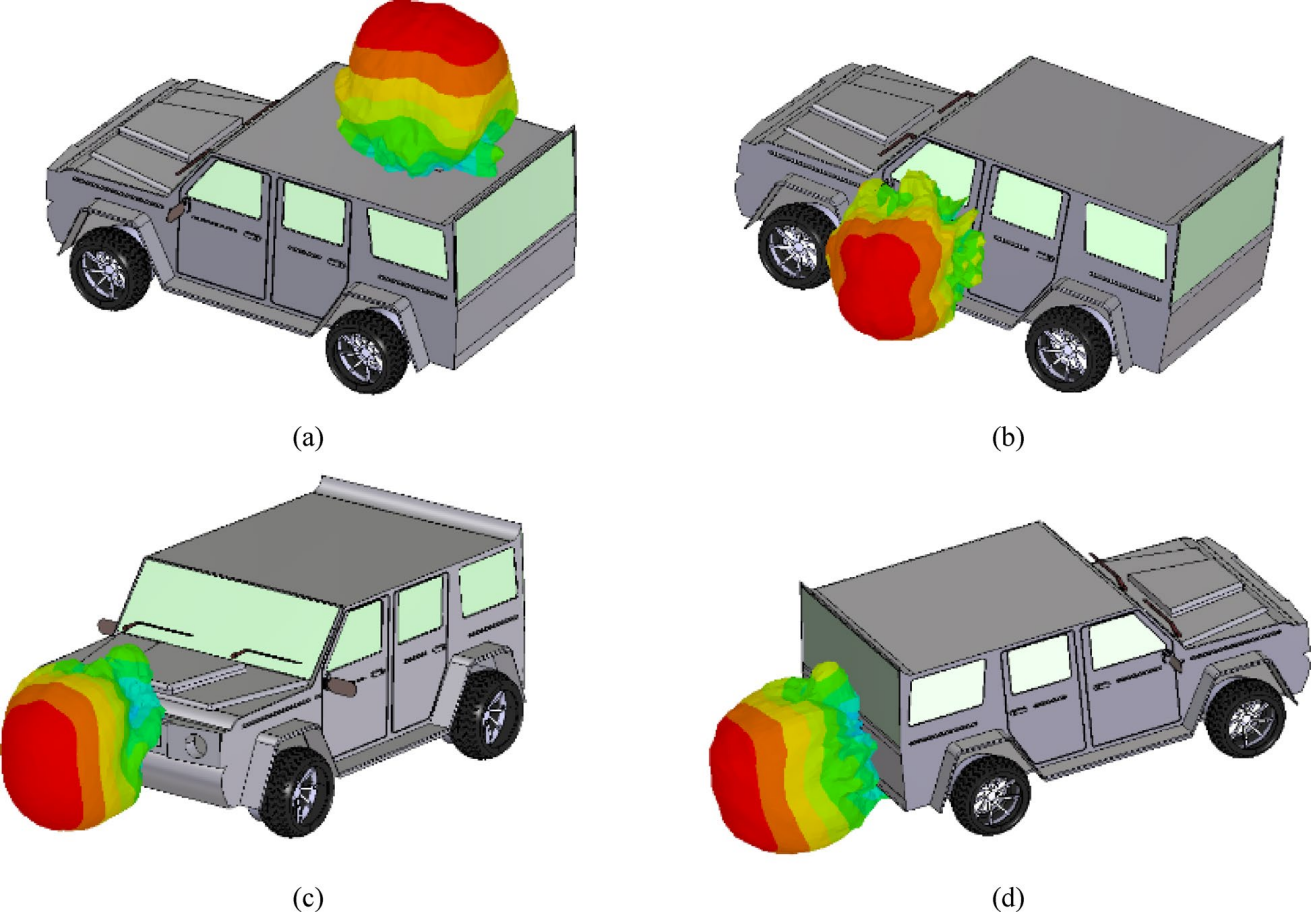
Radiation patterns of the designed eight-port MIMO antenna array at different locations on the vehicle: (**a**) Roof top, (**b**) Side door, (**c**) Front side, (**d**) Back side.

For V2V communication, the radiation performance of designed eight-port MIMO antenna array over the vehicle is presented in this section. In this work, CAD model of the vehicle is considered with its surface modelled as perfect electric conductor (PEC). The electromagnetic interaction of the antenna system with the vehicle is analyzed through CST Microwave Studio. The metallic body of the vehicle not only reflects but also scatter the EM waves. Due to the different surface characteristics of the vehicle body, the distinct radiation performance of the proposed antenna systems at each location is obtained. The designed eight-port MIMO antenna array is installed on roof top, side doors, front side, and back side of the vehicle. Here, the separation between antenna system and the vehicular surface is chosen as 2 mm. The proposed antenna system has nearly stable radiation pattern at each location of vehicle body (Fig. 17).

### V2V performance and link margin

**Fig. 18:**
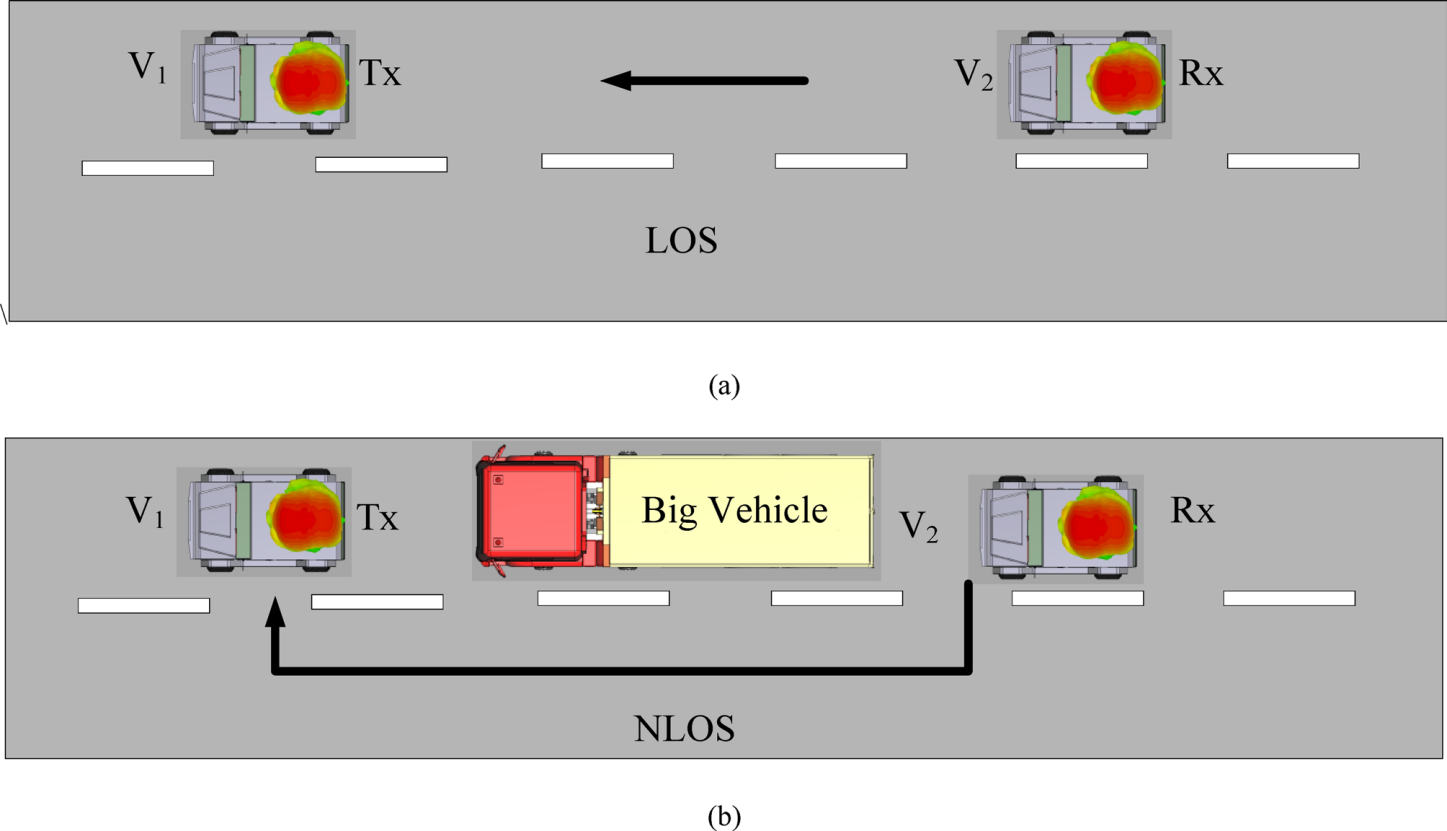
(**a**) LOS scenario, (**b**) NLOS scenario.

To examine the communication capacity of the proposed V2V millimeter-wave MIMO antenna array, the link budget analysis is performed in two different scenarios (LOS and NLOS) at its operating frequency. Here, to study the path loss and link margin for the proposed antenna system, communication between the antenna systems installed over the roof top of the vehicles is considered. The proposed antenna system has a half-power beamwidth (HPBW) of nearly 60°, producing a moderately focused beam that is oriented perpendicular to the antenna’s plane. When mounted on a roof, this beam spreads horizontally across the road surface, ensuring optimal coverage for approaching vehicles. The moderate HPBW of 60° minimizes vertical energy loss, which is often a problem with high-gain antennas that have a narrow HPBW. As per 3GPP release-14 in^41^, the effective distance for highway communication is approximately 320 m with a latency of 100 ms. Thus, the path losses and link margins of the designed antenna systems are evaluated up to 400 m in the present work. The environment setup for the evaluation of the path loss and link margin is shown in Fig. 18 for LOS and NLOS scenarios. In the LOS scenario, two vehicles V1 and V2 are in LOS and LOS-ABG path loss model is considered. While in the NLOS scenario, a big vehicle restricts the LOS communication between the two vehicles. In this case, the multipath signals results in the signal reception. The different parameters required for path loss and link margin are tabulated in Table 1.

**Table 1:** Parameters for path loss and link margin calculations.

Environment		
Operating frequency (GHz)	28	
*T*_*x*_/*R*_*x*_ Gain (dBi)	12.2	
*T*_*x*_/*R*_*x*_ Height (m)	1.5	
Parameters	LOS	NLOS
*R*_*x*_ Height (m)	1.5	1.5
PLEn	2.5	5.4
*σ*	3.5	14.8
*α*_*LOS*_	1.25	0.319
*α*_*ABG*_	1	2.8
*β*_*ABG*_	55	46.7
*γ*_*ABG*_	1.7	1.9
*σ*_*ABG*_	2.9	4.3
Signal		
Bit error rate (BER)	10^−6^	
*E*_*b*_/*N*_0_ (dB)	9.6	
Coding gain (dB)	0	
Fixing deterioration (dB)	2.5

1. LOS Scenario
  The V2V LOS communication occurs through direct LOS and ground reflected signals. In this case, there is a direct communication between the *T*_*x*_ and *R*_*x*_ vehicles is considered. The proposed MIMO antenna array is installed on roof top of the vehicles, where height of *T*_*x*_ and *R*_*x*_ are selected as 1.5 m. The path losses of LOS-scenario are computed for three large scale path loss models such as Friis free space path loss (FS), Stanford University Interim (SUI) and alpha–beta-gamma (ABG) models using the methods described in^42,43^. The three path losses are shown in Fig. 19a. The LOS-ABG model is considered for the calculation of link margin for LOS scenario. It is clear from the figure that the path loss in LOS-ABG model varies from 86 to 105 dB with an increase in the distance from 5 to 400 m. Considering the path loss from LOS-ABG model, link margins for different bit rates at the operating frequency of 28 GHz for LOS scenarios are plotted in Fig. 19b. In the analysis, the bit rates are varied from 1 Mbps to 50 Gbps. To have a reliable V2V communication link, 0 dB link margin is selected as a reference in the present work. The designed MIMO antenna array can provide 50 Gbps of data for nearly 200 m. The distance of the reliable communication link increases with a decrease in data rates.

**Fig. 19:**
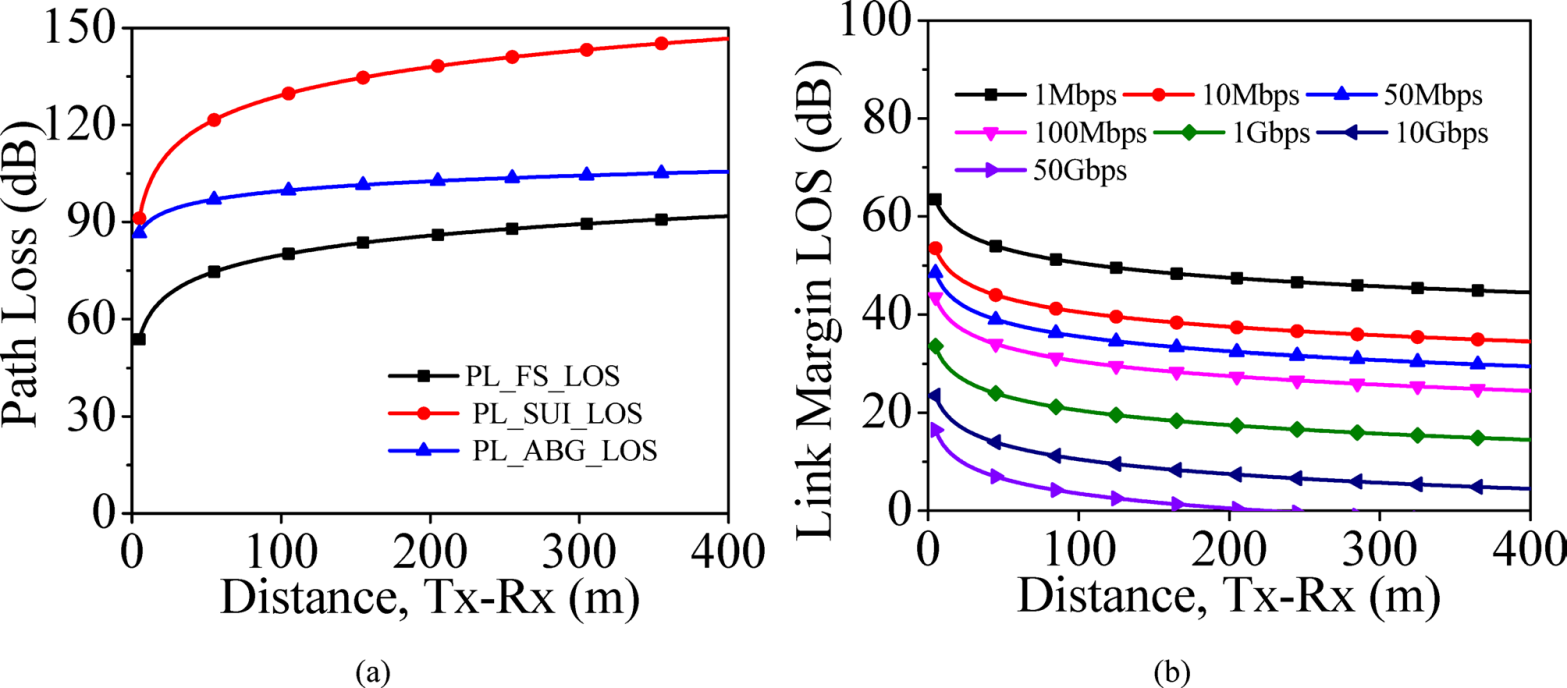
Performance in LOS scenario: (**a**) Path loss, (**b**) Link margin.
2. NLOS Scenario
  In the NLOS scenario, the other larger vehicles obstruct the communication between the *T*_*x*_ and *R*_*x*_. Here, the multipath channel results in the reception of the signals at the receiver end. Modified Friis free-space (FS-Mod) path loss, modified Stanford University Interim (Mod-SUI) and NLOS-ABG models are used for the calculation of the path losses in NLOS-scenario and are plotted in Fig. 20a. As there are other vehicles obstruct the channel between the *T*_*x*_ and *R*_*x*_, the path losses for NLOS scenario are larger than the LOS scenario. In NLOS-ABG model, the path loss varies from 93 to 147 dB as the distance ranges from 5 to 400 m. The link margin for the proposed millimeter-wave MIMO array is evaluated with the usage of NLOS-ABG model. Figure 20b shows the link margin for NLOS scenario for different bit rates at the operating frequency of 28 GHz. For a reliable communication link, the designed MIMO antenna array can provide 50 Gbps of data for nearly 70 m in NLOS scenario. Hence, the communication performance of the designed millimeter-wave MIMO antenna array depends upon LOS and NLOS scenarios.

**Fig. 20:**
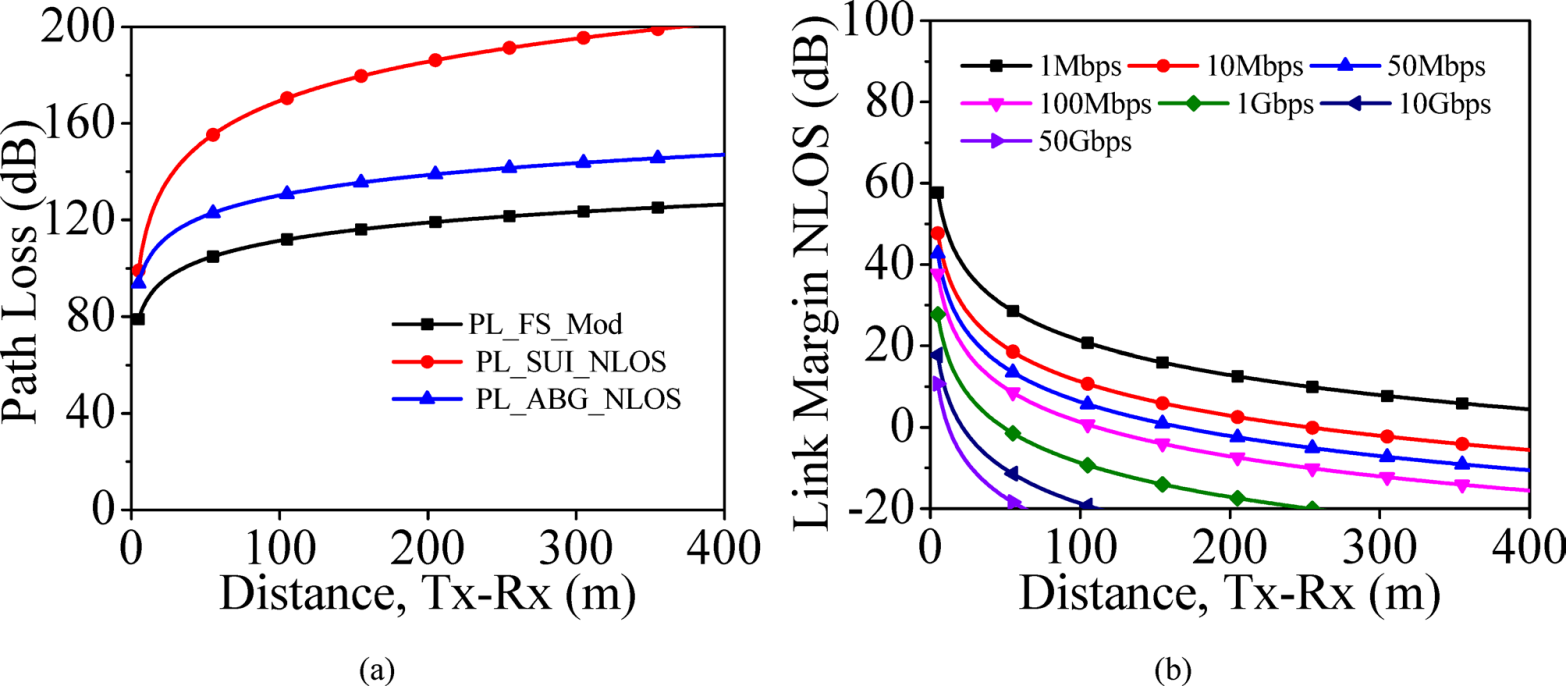
Performance in NLOS scenario: (**a**) Path loss, (**b**) Link margin.

## Comparison

To demonstrate the merits of the proposed millimeter-wave MIMO antenna array, it is compared with the other reported MIMO antennas in the literature. The performance comparison is presented in Table 2. Compared to the MIMO antenna designs presented in^22–24^, the proposed antenna possesses eight-port array configurations. Each port comprises of four HMSIW antenna elements. The antennas presented in^26–30^ have MIMO array configurations. However, all of them have lesser number of ports compared to designed MIMO antenna array. Moreover, the designed antenna array not only possesses eight port 2 × 2 array configurations, but also exhibits high inter-port isolations (> 30 dB) and higher gain (12.2 dBi). The proposed design methodology is further extended to eight port 4 × 4 MIMO antenna array configurations with a gain 17.4 dBi. The features of the proposed MIMO antenna array are summarized as follows:

**Table 2:** Comparison with other reported MIMO antennas.

Refs	*N*	Array (Yes/No)	No. of elements in each array	Operating freq. (GHz)	Size (${\lambda }_{0}^{2}$)	Ant. height (${\lambda }_{0})$	Iso. (dB)	Gain (dBi)	PL/LM (Yes/No)
^22^	6	No	–	25.5	1.28 × 2.04	NM	> 20	9.0	Yes
^23^	8	No	–	26	4.68 × 4.68	0.069	> 26	8.6	No
^24^	12	No	–	28	4.95 × 4.95	4.95	NM	6.0	No
^26^	2	Yes	4	38	2.46 × 4.93	0.031	> 22	12.8	No
^28^	4	Yes	2	28	4.07 × 4.07	0.037	> 20	7.9	No
^29^	4	Yes	4	28	2.87 × 5.45	0.047	> 30	12.5	No
^30^	4	Yes	4	30	2.0 × 1.0	0.31	> 15	10	No
Prop	8	Yes	4	28	5.6 × 5.6	0.023	> 30	12.1	Yes

*N*: No. of ports, ${\lambda }_{0}$: Wavelength at the center frequency,* Iso*. Isolation,* NM* Not mentioned,* PL* Path loss,* LM* Link margin.

(i) The proposed MIMO antenna array employs an octagonal arrangement of HMSIW elements, which enables compact geometry and high inter-port isolation (> 30 dB) without requiring complex decoupling structures.
(ii) The antenna achieves high gain (12.2 dBi), high FTBR of 30 dB, and low ECC across all ports, demonstrating its suitability for V2V millimeter-wave communication.
(iii) The design maintains a low profile and planar form factor, facilitating seamless integration into automotive platforms. Moreover, the designed MIMO antenna array can be re-designed to other frequencies of lower millimeter-wave spectrum by varying the dimensions of HMSIW cavity resonators.

## Eight-port MIMO millimeter-wave antenna array with 4 × 4 configuration

**Fig. 21:**
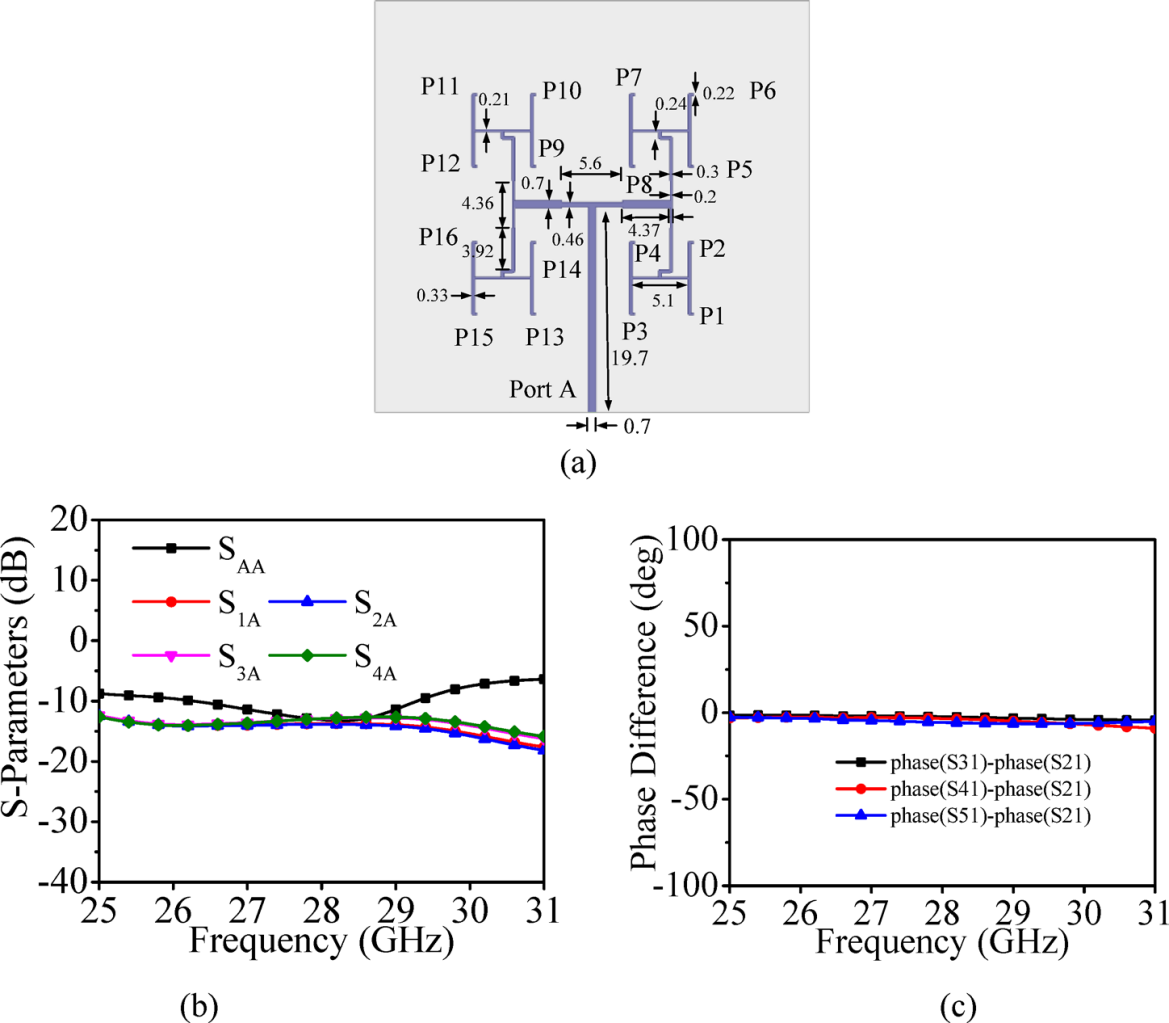
(**a**) 4 × 4 Feed network for the HMSIW unit element array (all dimensions are in mm), (**b**) Its simulated S- parameters, (**c**) Their phase differences.

To further enhance gain and radiation characteristics, the eight-port MIMO antenna design, originally based on a 2 × 2 array, is extended to a 4 × 4 array configuration. Initially, a 4 × 4 feed network is designed, which is further utilized for the design of the UEA, which contains sixteen HMSIW antenna elements. Figure 21a represents 4 × 4 corporate feed network for the HMSIW UEA. It comprises of several two-stage T-junction power dividers based corporate feed network. The S-parameters for the corporate feed network are plotted in Fig. 21b. The values of S_1A_, S_2A_, S_3A_ and S_4A_ are − 12.60 dB, − 12.84 dB, − 13.12 dB and − 13.02 dB, respectively demonstrates nearly equal power divisions at the output ports. The reflection coefficient at Port A is lower than − 12.2 dB, indicating good impedance matching. Figure 21c illustrates phase differences between the various output ports of the power divider, which are nearly identical at the operating frequency of 28 GHz.

**Fig. 22:**
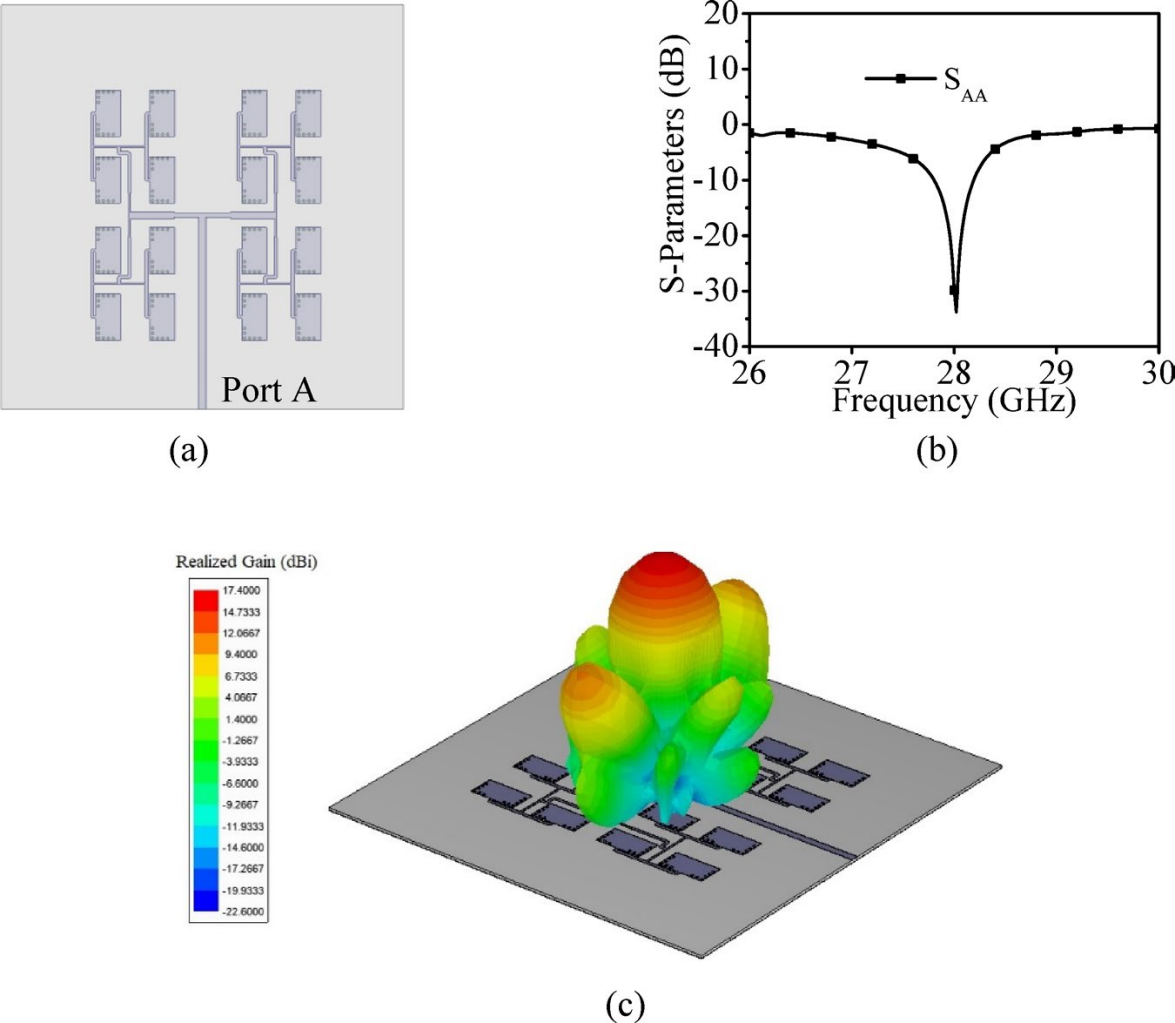
(**a**) Configuration of unit element array with 4 × 4 array pattern, (**b**) Its simulated S_AA_-parameters, (**c**) Three-dimensional far-field radiation pattern.

**Fig. 23:**
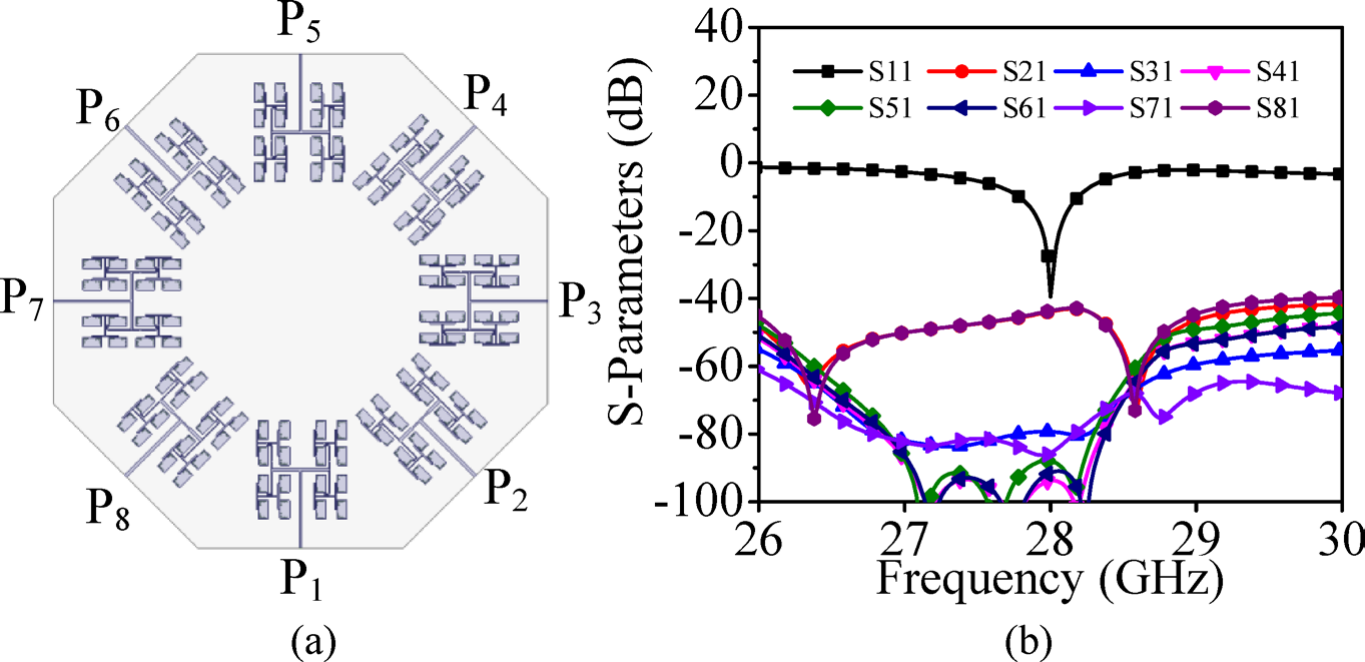
(**a**) Eight-port millimeter-wave MIMO antenna array with 4 × 4 array pattern for V2V communication applications, (**b**) Its simulated S-parameters.

To develop the high gain eight-port MIMO antenna array with 4 × 4 array pattern, a UEA at 28 GHz is designed using 4 × 4 corporate feed network. The configuration of unit element array, its simulated S_AA_-parameters and three-dimensional far-field radiation pattern are shown in Fig. 22. The UEA radiates at 28 GHz with a high peak realized gain of 17.4 dBi. Further, to design the eight-port millimeter-wave MIMO antenna array with 4 × 4 array pattern for V2V communication applications, eight-unit element arrays are placed across the edges of a regular octagon as shown in Fig. 23a. The simulated S-parameters of eight-port millimeter-wave MIMO antenna array with 4 × 4 array pattern are plotted in Fig. 23b. It can be observed that designed MIMO antenna array radiates at 28 GHz with the inter-port isolations better than 40 dB in the entire operating band.

## Conclusion

An eight-port millimeter-wave MIMO antenna array for V2V communication has been presented in this paper. The designed MIMO antenna array utilizes 2 × 2 HMSIW array as a unit element. The 2 × 2 HMSIW array is fed through a two-stage in-phase T-junction power divider. It utilizes the dominant TE_110_ mode of HMSIW cavity resonator to obtain radiations at 28 GHz. The high peak gain, high inter-port isolations, excellent radiation characteristics and good MIMO performance make the design MIMO antenna array a suitable candidate of V2V communication systems that operates at 28 GHz. The link budget analysis is performed with the evaluation of path loss and link margin. Moreover, the designed MIMO antenna array can be easily re-designed to any other frequency of lower millimeter-wave spectrum by varying dimensions of HMSIW cavity resonator. The design concept of the eight-port MIMO antenna array based on a 2 × 2 configuration is further extended to a 4 × 4 array pattern to improve gain and radiation performance.

## Data Availability

The data used to support the findings of this study are included in the article.
